# Deep learning techniques for early detection and classification of leaf diseases in crops

**DOI:** 10.3389/fpls.2026.1790903

**Published:** 2026-04-21

**Authors:** Parvathaneni Naga Srinivasu, Vemuri Anirudh, Devarasetty Likhitha Kamali, Somesetty Lalitha Kumari, Sudharshan Reddy Chidirala, Muhammad Fazal Ijaz

**Affiliations:** 1 Amrita School of Computing, Amrita Vishwa Vidyapeetham, Amaravati, Andhra Pradesh, India; 2 School of Technology, Business and Hospitality Faculty, Torrens University Australia, Melbourne, VIC, Australia

**Keywords:** deep learning, explainable AI, leaf disease classification, plant disease detection, precision agriculture

## Abstract

**Introduction:**

Rapid population growth and climate change have intensified the need for sustainable agricultural productivity. Plant leaf diseases significantly impact the crop yield, quality, and food safety, necessitating accurate and automated detection methods.

**Methods:**

This study proposes a deep learning (DL)-based framework for automated detection and classification of tomato and soybean leaf diseases. The proposed framework is trained and evaluated over a large-scale datasets comprising 16,012 tomato leaf images and 6,410 soybean leaf images. Multiple convolutional neural network (CNN) models, including DenseNet121, MobileNetV2, and InceptionV3, are employed for classification. Object detection is performed using YOLOv12. To enhance interpretability, Gradient-Weighted Class Activation Mapping (Grad-CAM) is integrated. Furthermore, a novel Hybrid Attention-Based Stacking Ensemble Model is developed using ResNet152V2, VGG19, and EfficientNetB0, combined with Convolution Block Attention Module (CBAM) and spatial attention mechanisms.

**Results:**

The CNN models achieved classification accuracies of 97% for DenseNet121, 98% for MobileNetV2, and 99.94% for InceptionV3. YOLOv12 attained a mean average precision (mAP) of 99.5%. The proposed hybrid ensemble model achieved an accuracy of 99.18%, demonstrating improved feature learning through combined channel and spatial attention. Grad-CAM visualizations confirmed that the model effectively identifies the disease-relevant regions.

**Discussion:**

The results indicate that the proposed framework has attained a high accuracy, robustness, and interpretability for plant disease detection. The integration of attention mechanisms and explainable AI enhances model reliability and transparency. This framework shows a strong potential for the real-time agricultural monitoring, although further validation across diverse crops and real-world field conditions is required.

## 1 Introduction

Agriculture is fundamental to global food production and economic sustainability, supporting food security and livelihoods worldwide (World Bank, 2019). Agricultural production and supply chains continue to expand rapidly, and the importance of crops like the tomato is set to increase as the world’s population continues to grow (Godfray et al., 2010). It is also the most popular fruit and vegetable crop, and its rich contents of vitamins, minerals, and antioxidants, such as lycopene, have been associated with the prevention of human diseases and promotion of health (Rao and Agarwal, 1999). Yet, fruit and vegetable farming is threatened by numerous foliar diseases, such as early blight, Septoria leaf spot, bacterial spot, tomato yellow leaf curl virus (TYLCV), and tomato mosaic virus (ToMV) (Nawaz et al., 2022; Sujatha et al., 2025). These diseases cause a reduction in chlorophyll content and photosynthesis, and consequently, premature leaf dropping, smaller fruit size, and decreased yield and quality (Lin and Huang, 2024; Alirezazadeh, P., Schirrmann, M., and Stolzenburg, F. (2023)). If these diseases are allowed to spread unchecked, they can cause significant damage to crop yields, risking food security as well as causing turmoil in local and international markets. Therefore, early detection of leaf diseases is essential to apply control measures in time, reduce crop loss, and promote sustainable agriculture (Jackulin and Murugavalli, 2022).

Plant disease identification has traditionally been done manually through visual observation by agricultural experts. Although suitable for household gardening, these techniques are not only labor intensive and time consuming but also prone to human bias (Kaur and Garg, 2021). Environmental factors—illumination, humidity, and background clutter—and natural variations in leaf color, shape, and texture make diagnosis even more challenging (Wu et al., 2025). These constraints motivate the design of intelligent, automated frameworks that can achieve early and accurate detection of plant diseases in general field environments. Such systems can contribute decisively to pest and disease management, minimizing dependence on chemicals and increasing agricultural productivity.

Recent progress in artificial intelligence (AI) and deep learning has revolutionized precision agriculture by facilitating data-driven analysis and automation in the field (Lin and Huang, 2024; Alirezazadeh et al., 2023). Among them, convolutional neural networks (CNNs) (Kaur and Garg, 2021) have achieved superior performance over traditional machine learning methods by directly processing raw leaf images to learn hierarchical spatial and texture features rather than relying on handcrafted features. CNN-driven models like the DenseNet, MobileNet, and Inception architectures have also shown significant levels of accuracy and robustness in detecting multiple plant leaf diseases (Liang and Jiang, 2023; Liu et al., 2022). Moreover, the extension of CNNs with attention mechanisms allows models to focus selectively on the most informative parts of diseased leaves, enhancing both interpretability and accuracy of disease identification and localization (Wang et al., 2024). These advances represent a first step toward scalable, explainable, high-performing AI systems for solving high-impact agricultural challenges. However, there are still several practical barriers to real-time implementation of DL models in the field. Most state-of-the-art architectures are computationally expensive and have high memory requirements, which restrict their use on mobile or edge devices (Too et al., 2019). In addition, environmental factors such as illumination variation, mild overlapped symptoms, and background interference may degrade model reliability on field images. Furthermore, the black-box nature of DL systems hinders their adoption, as farmers and agronomists need clear and interpretable models for decision-making they can trust (Samek et al., 2017). These challenges highlight the potential benefits of lightweight, flexible, and explainable DL architectures that balance accuracy, interpretability, and computational efficiency.

Although light detection and ranging (LiDAR) and AI algorithms for plant disease detection have improved, several research gaps remain (Kamilaris and Prenafeta-Boldú, 2018). State-of-the-art CNN architectures, while highly accurate on benchmark datasets, have limited generalization capability when applied to field-captured images with illumination changes, shadowing, and background variations (Barbedo, 2019). Due to the computational inefficiency of DL models, their application to mobile or low-power devices used in field environments remains limited. Also, the opacity of traditional CNN models reduces user confidence since the end user cannot see why a certain prediction is made. As a result, biased learning can lead to poor detection performance on minority disease classes. Finally, investigation of hybrid and attention-based CNN models that simultaneously enhance robustness, transparency, and computational efficiency is still underdeveloped for practical agricultural applications (Arrieta et al., 2020).

The current study introduces an integrated explainable deep learning–based framework for image-level disease classification, lesion localization, and model interpretability within a unified end-to-end pipeline. Pretrained hybrid CNN architectures are used for the extraction of hierarchical features to perform multi-class disease classification. Then, a YOLOv12 detector is used to localize the infected area by predicting bounding boxes, achieving spatial output for the diagnostic result. Furthermore, Gradient-Weighted Class Activation Mapping (Grad-CAM)–based explainable artificial intelligence (XAI) is included for producing attention maps reflecting discriminant regions that contributed to making model predictions. The combination of CNN-based classification, YOLOv12 localization, and gradient-based interpretability creates a coherent classification–localization–interpretability framework for enabling precision agriculture and smart crop monitoring solutions.

- A robust disease prediction by employing hybrid attention-based ensemble model.
- Integration with YOLOv12 detection for the spatial localization of lesions.
- Interpretability by guided Grad-CAM for increased transparency and better understanding of features.

Many YOLO-based models have been newly proposed to enhance the performance of object detection in the field of agriculture. For example, Li et al. (2025) proposed Succulent-YOLO based on the integration of attention mechanisms for better feature extraction in plant monitoring. In the same vein, You et al. (2025) introduced Rose-Mamba-YOLO by adding state-space modeling principles to reinforce long-range feature representation in the greenhouse. Moreover, few recent YOLO-based variants have dabbled with lightweight detection networks, enhancing feature pyramids for small-object detection, and domain-adaptation methodologies to bolster robustness under severe environmental conditions. Some good examples are Rose-YOLO, Mussel-YOLO, FP-YOLO, RBL-YOLO, and UDD-YOLO presented in recent works on agricultural and environmental monitoring. Yet these methods are in most cases for enhancing detector architecture. In contrast, we consider unification of classification, localization, and explainability, combining detection with interpretability of the model. The advent of AI has transformed a variety of industries beyond its early applications in medical diagnosis and data processing, including agriculture, especially with regard to the automated identification of crop diseases. In plant pathology (Tugrul et al., 2022; Suresh Manic et al., 2024), deep learning and AI-based systems are increasingly being utilized to overcome the limitations of human visual inspection under varying lighting conditions, noise in the background, and slight variations in symptoms, enabling swift and precise identification of disease at the leaf level. Precision agriculture research now utilizes CNNs, vision transformers, and hybrid models to deal with big datasets, achieving robustness in real-world conditions and facilitating execution on portable devices. Research indicates that these sophisticated systems are able to greatly enhance yield protection and food security by facilitating timely intervention Batool and Byun (2025).

A sequential approach to image analysis and optimization has been reported to enhance the analytical performance (Badiger and Mathew, 2023), and deep neural frameworks for enhancing disease segmentation and recognition efficiency. Their model combined the segmentation methods of Particle Swarm Optimization, Competitive Swarm Optimization, JAYA, and Gradient JAYA-Golden Search Optimization to enhance infected region extraction. A variant of deep architecture, DbneAlexNet, was utilized for the classification of different diseases of tomato leaves post-segmentation to provide strong classification across various disease classes. The method proved that optimization-based segmentation coupled with DL improves both accuracy and model robustness for real-world agricultural applications.

The development of deep learning for plant disease detection has seen many variations and permutations of architectural innovations and deployment schemes in recent years. Paul et al. (2023) demonstrated the practical feasibility of transferring laboratory models to field applications by training a lightweight CNN on more than 30,000 tomato leaf images. On their method, they performed an extensive data augmentation to improve generalization and successfully published the system through TensorFlow Lite on the web and Android, allowing for real-time detection and classification of diseases in the fields.

Comparative studies have consistently reported that deep learning outperforms traditional machine learning. In a thorough assessment, Nikith et al. (2023) CNN-based models were compared with traditional classification algorithms such as Support Vector Machines and K-Nearest Neighbors for the task of soybean leaf disease classification. The results showed that CNNs significantly outperformed traditional methods because CNNs are capable of learning discriminative features in both the spatial domain and the texture domain from raw image data without crafting any hand-engineered features. This study highlighted the paradigm shift toward deep learning–based architectures, which can efficiently classify leaf diseases.

Several researchers have further developed baseline models based on classical CNN architectures for agriculture (Anandhakrishnan and Jaisakthi, 2022). exploited the architecture of AlexNet and LeNet to design a deep convolutional network for multi-tomato leaf disease identification. Based on the large PlantVillage dataset and using ReLU activations, max pooling, and fully connected layers, the hierarchical architecture captured highly complex spatial diseased leaf regions and outperformed conventional machine learning methods in classification.

Architectural improvements were made to achieve a good trade-off between accuracy and computational cost. Zhou et al., 2021 proposed a novel rearrangement of RDN for agricultural images. By reorganizing RDBs as well as facilitating gradient flow, we created a more efficient network that introduces even higher levels of feature reuse and relies on fewer parameters yet achieves comparable accuracy on tomato leaf disease classification. The reformulated model reduced the computational burden and achieved good performance, showing great potential for practical application.

The combination of transfer learning with custom model architectures has proved to be successful in the case of resource-limited applications of the technique (Shehu et al., 2025). Shanthi et al. (2024) proposed a CNN model by integrating data augmentation and transfer learning for efficient feature extraction. The multi-layered framework successfully represented various disease patterns and textures to deliver high-precision infected leaf recognition under different scenarios. The miniaturized architecture was for deployment in agricultural environments with limited computational resources.

Recent advancements in the YOLO family have significantly improved real-time agricultural diagnostics. Ramos and Sappa (2025) conducted a benchmarking study comparing YOLOv8 to YOLOv12 for tomato leaf disease detection using the Tomato-Village dataset. Their results showed that YOLOv11 offers the best accuracy-latency trade-off, while YOLOv12n is more suitable for lightweight edge-device deployment. The study also highlighted performance limitations in YOLOv9 and efficiency improvements introduced through YOLOv12’s attention mechanisms, establishing an important performance benchmark for future research.

The study has designed an efficient and compact CNN based on GoogLeNet, which realizes the enhancement of feature extraction ability with low computational cost (Dai et al., 2023). with spatial pyramid pooling to promote multi-scale feature extraction. The enhanced architecture, named GoogLeNet-EL, retained high performance at significantly lower computational demands. It showcased the capabilities of lightweight CNNs to provide accurate and efficient disease recognition, which allows for adoption in large-scale agricultural systems and on-device applications. DL has shown great promise for plant leaf disease detection with better feature extraction and classification capabilities than traditional methods. The ToLeD: Tomato Leaf Disease Detection using the CNN model is based on a multi-layered CNN architecture, among which three are convolutional layers, two are pooling layers, and one is a dense layer to automatically detect tomato leaf disease. The model achieved better performance than other existing DL models, but they highlighted that the classification across disease categories is not stable, and the generalization on crops other than tomato is limited.

Then, a Restructured Deep Residual Dense Network (RRDN) was proposed to increase feature reuse with better representation learning and promote disease recognition. This method, although powerful, still remained tailored to one particular dataset and was not flexible enough to be used in other scenarios, i.e., different environmental and crop conditions, which limits the applicability in real-world agriculture.

A special kind of CNN with ResNet-50 and data augmentation techniques was also used for the purpose of improving generalization and preventing overfitting. Another study performed data augmentation and used a CNN and Inception V3 model for classification. Although augmentation has greatly enhanced the ability of models to manage the diversity of leaf images, it also increases their computational complexity and training requirements, which limits their suitability for resource-constrained or remote deployment environments.

Several recent works have investigated DL-based solutions for enhancing the detection and classification rates of tomato leaf diseases. In the paper on tomato leaf disease detection and classification using CNN, several architectures such as CNN, VGG16 (Tariq et al., 2024), ResNet, and InceptionV3, among others, were used in combination with sophisticated data augmentation methods such as GANs, DCGAN, WGAN, and neural style transfer. These enhancements led to better feature representation robustness and model generalization. But the model was restricted to only tomato crops, and its reliability was compromised in the presence of real-world noise and unknown disease cases.

In another study, Tomato Leaf Disease Detection Using CNN, they proposed CNN architectures as a baseline and AlexNet and VGG16 as the composite classifier, with the addition of image preprocessing and data augmentation, to improve the classification performance of the different packages, which contained different tomato diseases such as early blight, leaf mold, and yellow leaf curl virus. The proposed method achieved superior performance with an improved ability to discriminate between disease types but had limited generalization ability to other crop species and real-world environments due to environmental factors.

Additionally, a hybrid model combining CNN, AlexNet, VGG16, LeNet, and ResNet50 was utilized for transfer learning and data augmentation in tomato leaf disease detection Using DL techniques for complete disease detection and identification. Although these deep networks achieve powerful learning, they require a large amount of labeled data and computational resources, which limit their applicability for precision agriculture on the field or in a developing country.

This paper is organized as follows: Section 2 presents the methods and materials used in the current study, existing DL classifiers, and the proposed Hybrid Attention-Based Ensemble Model, and Section 3 presents the results and analysis. 4 presents the discussion on the observed results. Finally, Section 5 concludes the study and discusses future research directions toward sustainable agricultural AI systems.

## 2 Materials and methods

The current section of the manuscript presents the details of the datasets that are being used and various DL models that are being evaluated as part of the study.

### 2.1 Dataset description

The datasets employed in this study were obtained from the Kaggle open-source platform, which contains highly structured and well-labeled images suited for DL in agriculture. This work employed the Tomato Leaf Disease Dataset and the Soybean Leaf Disease Dataset. Both data sets contain high-quality RGB images of leaves from different plants, both diseased and healthy, as given in **Tables 1**, **2**. To keep the training process consistent and to extract features efficiently by the CNNs, each image was normalized to a fixed size of 224 × 224 × 3 pixels.

**Table 1:** Summary of the datasets used in this study.

Dataset	Source	Total images	Classes	Class categories	Image size
Tomato Leaf Disease Dataset	PlantVillage (Kaggle)	16,012	10	Bacterial spot, Early blight, Late blight, Leaf mold, Septoria leaf spot, Spider mites, Target spot, Yellow leaf curl virus, Mosaic virus, Healthy	224 × 224 × 3/299 × 299 × 3
Soybean Leaf Disease Dataset	Soybean-Latest (Kaggle)	6,410	3	Caterpillar, Diabrotica speciosa, Healthy	224 × 224 × 3 / 299 × 299 × 3

**Table 2:** Class-wise distribution of tomato and soybean leaf disease datasets.

Tomato classes	Number of images
Tomato_Bacterial_spot	2,127
Tomato_Early_blight	1,000
Tomato_Late_blight	1,909
Tomato_Leaf_Mold	952
Tomato_Septoria_leaf_spot	1,771
Tomato_Spider_mites_Two_spotted_spider_mite	1,676
Tomato:Target_Spot	1,404
Tomato:Tomato_YellowLeaf:Curl_Virus	3,209
Tomato:Tomato_mosaic_virus	373
Tomato_healthy	1,591
Soybean classes	Number of images
Caterpillar	3,309
Diabrotica speciosa	2,205
Healthy	896

#### 2.1.1 Tomato leaf disease dataset

**Figure 1:**
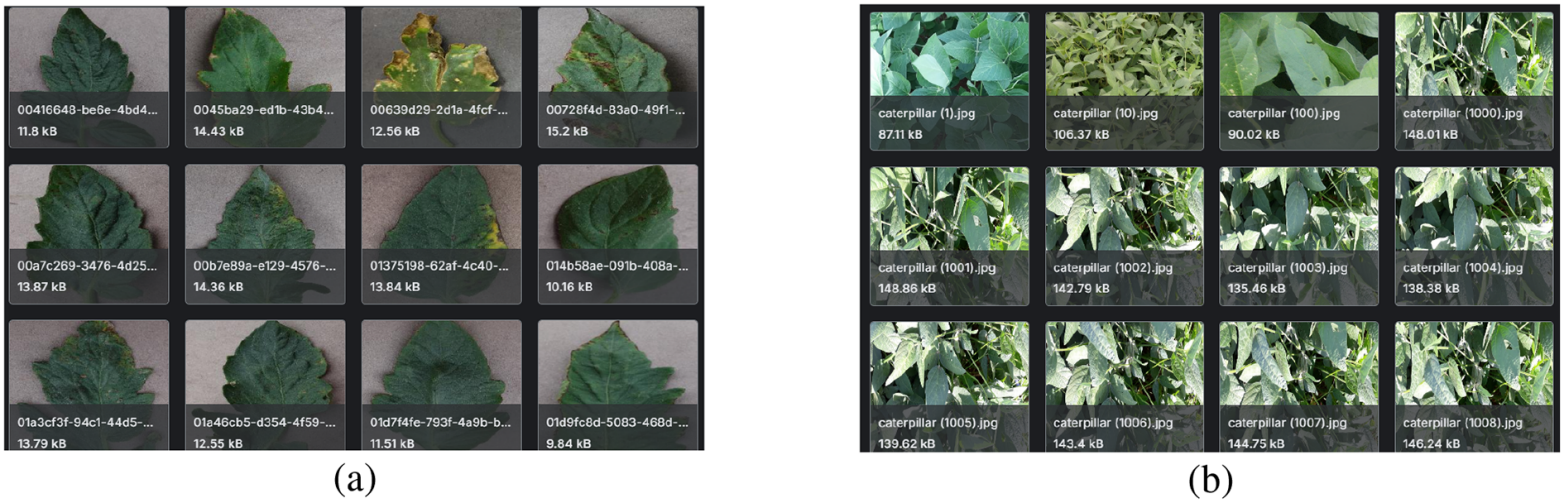
Image samples from the leaf disease datasets: **(a)** Tomato Leaf Disease Dataset, **(b)** Soybean Leaf Disease Dataset.

The *tomato leaf disease dataset* in this study was fetched from the public PlantVillage dataset available on Kaggle (Rex, 2019), which contains a total of 16,012 images under ten different classes of diseased and healthy leaves (**Figure 1a**). The class names are *Tomato_Bacterial_spot*, *Tomato_Early_blight*, *Tomato_Late_blight*, *Tomato_Leaf_Mold*, *Tomato_Septoria_leaf_spot*, *Tomato_Spider_mites_Two_spotted_spider_mite*, *Tomato_Target_Spot*, *Tomato_YellowLeaf_Curl_Virus*, *Tomato_mosaic_virus*, and *Tomato_healthy*. All the images depict the visible symptoms of tomato leaf diseases such as discoloration, texture variations, and structural deformations. The dataset exhibits substantial intra-class and inter-class variability, enabling DL models to effectively discriminate among different disease types and infection stages.

#### 2.1.2 Soybean leaf disease dataset

The *Soybean Leaf Disease Dataset*, applied in this research, was acquired from the Soybean-Latest dataset on Kaggle, which is publicly accessible (Patil, 2024) and has a total of 6,410 images among three categories: *Caterpillar*, *Diabrotica Speciosa*, and *Healthy* (**Figure 1b**). Here, the soybean leaves, which have infestations, were infected with a pest and also include a damaged leaf and an undamaged one under natural conditions and field-sampled conditions, respectively. The distribution of class is caterpillar with 3,309 images, Diabrotica speciosa with 2,205 images, and healthy with 896 images of leaves. All images were resized to 224 × 224 × 3 pixels to maintain architectural consistency across all backbone networks. A key aspect that makes the soybean dataset advantageous for this study is its capacity to test the model in detecting pest-infested leaf damage, in contrast to disease-related visual symptoms.

### 2.2 Data preprocessing

Preprocessing is an essential stage in agricultural image analysis for DL to make the images consistent, highlight the useful visual information, and eliminate the noise. Here, preprocessing was performed to eliminate the effects of illumination, resolution, orientation, and complexity of the background, all of which will distort model convergence and predictive accuracy if left uncorrected. This procedure included resizing the images, rescaling the pixel intensity values, and enhancing the contrast of the images to highlight the features related to the diseases. The result of this simplified preprocessing approach was a much cleaner and more homogeneous data set, which led to more stable training and better generalized performance for all model components.

#### 2.2.1 Label encoding

Since deep neural network architectures work on numbers, the categorical class labels were converted first to integer indices, and then, at the time of model training, these integer indices were transformed into one-hot encoded vectors (Javidan et al., 2024). Such an encoding scheme is indeed consistent with the softmax activation function and yields equal propagation of gradients to all nodes in the output layer. The transformation is precisely mathematically defined as Equation 1.

(1) $$y_{\text{encoded}}=\text{OneHotEncode}(y),\;y\in \{0,1,\ldots ,C-1\}$$

where *C* is the total number of disease classes.

#### 2.2.2 Image standardization

All images were resized during preprocessing to the selected backbone architectures’ input size (either 224 × 224 or 299 × 299 pixels), so the shape of the input tensor was fixed and consistent for all models. Then the pixel intensities were normalized to the [0, 1] range by using the min–max scaling as Equation 2.

(2) $$I_{\text{scaled}}=\frac{I}{255}$$

In the case of dataset-wise standardization (Chen et al., 2023), one more normalization step was carried out as Equation 3.

(3) $$I_{\text{normalized}}=\frac{I-\mu }{\sigma }$$

where 𝜇 and 𝜎 are the global dataset mean and standard deviation. Normalization (Das et al., 2025) compensates for differences in brightness and exposure among samples, thus improving model robustness and feature stability.

#### 2.2.3 Adaptive contrast refinement

In order to intensify subtle disease-related visual signals, adaptive gamma correction (Harakannanavar et al., 2022) with the gamma value randomly sampled from the interval [0.8, 1.2] was used. The transformation is mathematically defined as shown in Equation 4.

(4) $$I_{\text{enhanced}}=I_{\text{scaled}}^{\gamma }$$

Gamma values less than 1.0 make the image brighter, and more than 1.0 makes the image darker. This selective contrast enhancement (lesion contrast and background homogeneity) makes lesions more discernible and also maintains natural appearance and can thus improve the feature extraction (Anitha and Srinivasan, 2022) capability at the next convolutional layer.

### 2.3 Data augmentation

**Figure 2:**
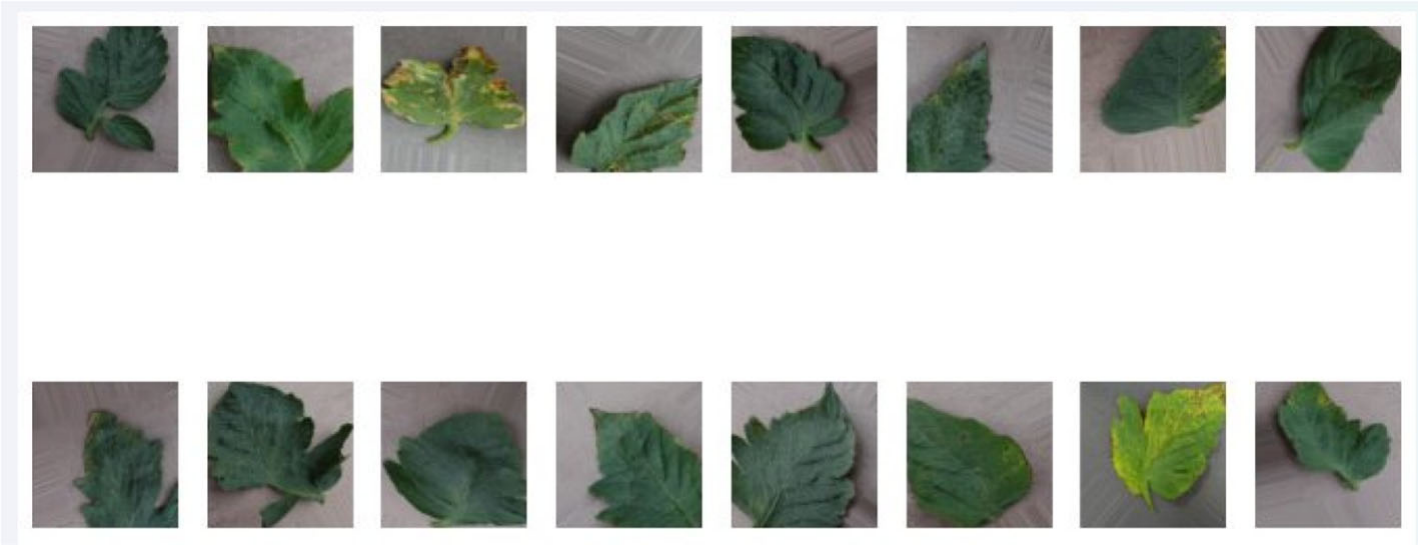
Sample images after augmentation.

In order to improve the generalization of the model and counteract overfitting, the online data augmentation is performed with controlled randomness (Antwi et al., 2024). This augmentation pipeline consisted of spatial and photometric transformations that mimicked diverse real-world scenarios and helped in generalizing the model against unseen data. Geometric augmentations included rotation (± 40°) to introduce variability in viewpoint, width and height shifts (± 30%) to simulate misalignment of camera, shear transformations (± 0.3) (Wang, 2024) to introduce affine distortions, and zoom (± 30%) to enforce scale invariance and isotropy of space. In addition, the horizontal and vertical flips were adopted to learn the natural symmetries of many objects and scenes so that the network can better identify the mirrored or inverted patterns. Photometrically, brightness corrections ([0.6–1.4]) were applied to imitate varying illumination, whereas channel shifts (± 50) were used to achieve robustness against sensor noise and/or chromatic aberrations (**Figure 2**). For improved statistical independence of the input features, pixel intensities were whitened using ZCA whitening 18 (Shewale and Daruwala, 2023), which decorrelates the intensities and also well conditions the network by normalizing the feature covariance. Furthermore, feature-wise centering and normalization were applied so as to have the same feature distribution throughout the entire dataset, which is beneficial for maintaining consistent input statistics throughout training. Together, these transformations produce a more diverse synthetic dataset from which the network can learn to extract robust, invariant, and discriminative features that allow it to generalize well over arbitrarily complicated visual domains without overfitting to training data.

Fairness to comparison: the same augmentation setup is used for all classification backbones: DenseNet121, MobileNetV2, ResNet152V2, InceptionV3, and our hybrid ensemble. For the YOLOv12 detection architecture, only spatial augmentations (e.g., flipping, scaling, rotating) were allowed, and ZCA whitening and heavy photometric transformations were ignored in order to maintain bounding-box consistency. This unified, yet task-specific, augmentation strategy guarantees that the ensuing performance gap is attributable to architectural design, not preprocessing bias.

### 2.4 Feature extraction

Feature extraction plays an essential role in the automated classification of leaf diseases, which converts the raw image data into informative feature representations that contain characteristic pattern information of individual diseases. Conventional handcrafted techniques may not be suitable for multiscale pattern recognition in spatial and texture domains, the present work utilizes deep CNNs to perform feature extraction at multiple hierarchical levels (Das et al., 2025).

**Figure 3:**
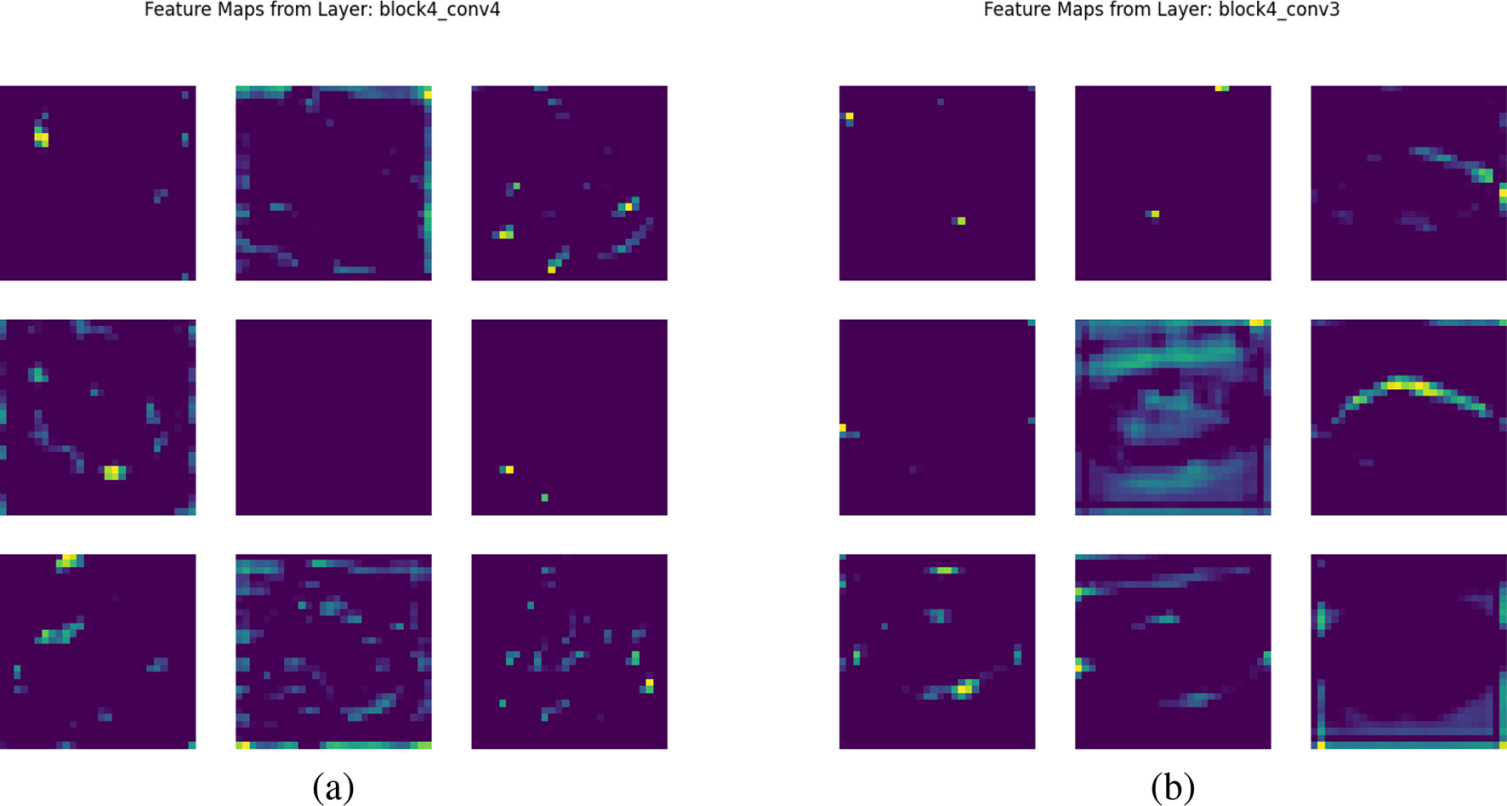
Feature maps for leaf images. **(a)** Soybean leaf. **(b)** Tomato leaf.

The preprocessed images were resized, normalized, and then sent to the well-known pretrained CNNs including DenseNet121, MobileNetV2, InceptionV3, and YOLOv12. Among them, DenseNet121 was mainly used for feature extraction from soybean and tomato datasets (**Figure 3**). Dense connectivity, which connects all layers to each other, promotes feature reuse and improves gradient propagation and alleviates the vanishing-gradient problem, and therefore it leads to good learning even for small datasets.

To eliminate non-informative background regions and highlight disease-related patterns, CBAM (Channel & Spatial Attention) (Woo et al., 2018) module in VGG19 and the Spatial Attention module in EfficientNetB0 were applied. The refined representation is obtained as Equation 5.

(5) $$F=M_{S}⊙(M_{C}⊙\phi (X;\theta ))$$

where M and $M_{S}$denote channel-wise and spatial attention masks, and ⊙ indicates element-wise multiplication.

The output feature maps are transformed to a one-dimensional vector using global average pooling (GAP) (Chollet, 2017) Equation 6 as follows:

(6) $$F_{\text{GAP}}(k)=\frac{1}{H\times W}\sum_{i=1}^{H}\sum_{j=1}^{W}F_{k}(i,j)$$

where H and W denote spatial resolution, and k is the channel index. To enhance the separability of classes, the pooled feature map was sent to a fully connected layer as Equation 7.

(7) $$Z=\text{σ}(WF_{\text{GAP}}+b)$$

where *W* and *b* are trainable parameters, and σ(.) is a ReLU activation. To improve prediction reliability, the probability outputs of the three models were combined using equal-weight soft voting. The ensemble probability was computed as the arithmetic mean of the individual model probability vectors (Zhou et al., 2021), as shown in Equation 8.

(8) $$P_{\text{ensemble}}=\frac{P_{R}+P_{V}+P_{E}}{3}$$

where $P_{R}$, $P_{V}$, $P_{E}$ are model outputs. The output prediction was achieved by softmax normalization as Equation 9.

(9) $$P(y=c|Z)=\frac{e^{Z_{c}}}{\sum_{k=1}^{C}e^{Z_{k}}}$$

where C is the total number of disease classes. Eventually, each image is encoded into a high-dimensional feature vector, which includes the most discriminative visual cues for the images, based on which we perform feature selection and classification.

### 2.5 Feature selection

Feature selection is the identification of the most informative features in the extracted representations that can significantly improve the efficiency and accuracy of the model. Deep learning models inherently perform implicit feature selection during training. But this work introduces additional explicit selection-based approaches for refining the learned feature space (Rehman et al., 2023). They used a GAP layer (Chollet, 2017) following the convolutional feature extraction to transform each feature map into one single value (Senthil Pandi et al., 2022). This method is an excellent summary of the spatial features in the feature map, and it also strongly decreases the number of parameters (Zhang et al., 2018). The GAP layer also removes redundant or irrelevant activations, which prevents overfitting and enhances the overall performance of the model.

The hybrid ensemble combines the features selected from different CNN backbones like ResNet152V2, VGG19, and EfficientNetB0 through weighted feature aggregation. The ensemble feature (Ali et al., 2024) fusion guarantees that complementary information of multiple architectures can be used to make final predictions. The CBAM and spatial attention modules add more sophistication to this approach by focusing more on features that were highly related to disease regions while deemphasizing background or non-informative regions.

Finally, the refined feature vector with the most discriminative and biologically valuable patterns is fed into fully connected dense layers for classification. This step allows us to train the classifier on a feature subset that is both small and informative, improving prediction accuracy, reducing computational effort, and making the model more interpretable.

### 2.6 Dataset splitting

The data was split in 80:10:10 ratio for training, testing, and validation subsets to attain sufficient training samples and also unbiased evaluation results. In detail, 80% of the images were employed for training the model to learn the feature representations, 10% for validation to adjust the hyperparameters and to avoid the overfitting problem, and 10% for testing to estimate the capability of the model to generalize the previously unseen data. We used a stratified splitting method to keep the class distribution in all sets as train, validation, and test. It made the model more stable and robust in testing. Stratified splitting was done using the train test split function from scikit-learn with shuffle set to True and random seed fixed to ensure exactly the same dataset splits for all the runs. The proportion of each class was kept the same in each subset to prevent sampling bias. A pie chart for each subset was drawn to make it clearer, as shown in **Figure 24**. There was no oversampling or synthetic class balancing; rather, the natural class imbalance was preserved to better represent realistic distributions of agricultural data.

For reproducibility, all data splitting and training were carried out with a fixed random seed of 42. The same seed was also always used for stratified splitting, model initialization, and augmentation pipelines to ensure deterministic behavior in repeated runs. This setup reduces random noise in the performance measure and allows for fair comparisons of different architectures.

We always use a fixed random seed throughout our experiments for deterministic runs. Thus, it produces the same splits (subsets) of the dataset and the same initial states (weights) of the model for every run, minimizing the variance. This regulated setting almost ensures the performance comparisons are never affected by noise.

Before splitting the dataset, we performed integrity checks to verify that there were no duplicated or shared images between the training, validation, and test sets. We conducted file-level comparisons and class-wise analysis to mitigate any possible data leakage and so enhanced the reliability of the benchmarking results.

In addition, to reduce the risk of data leakage, visual similarity and file level were applied to make sure that highly similar samples, duplicate leaves, or images from the same plant do not appear in different folds. This prevents potential overestimated performances due to memorization as opposed to generalization.

### 2.7 Model selection

These models, DenseNet121, MobileNetV2, ResNet152V2, InceptionV3, and YOLOv12, are considered baseline architectures for benchmarking in this work. The proposed contribution is a hybrid attention-based stacking ensemble model that combines several CNN backbones with attention mechanisms for better feature representation and classification. In this work, we have systematically investigated and evaluated a number of state-of-the-art deep CNN architectures for the challenging problem of plant leaf disease classification and prediction. The essence of the model selection process was to find an architecture that balances accuracy and computational efficiency, allowing the tradeoff among several important considerations, including high classification accuracy, computational efficiency, stable convergence in the training stage, and generalization capability when tested on different, unknown, heterogeneous agricultural data sets. Balancing these considerations is especially important in agricultural image analysis, where models must generalize well to field data that are often collected under variable conditions using different capture devices.

To overcome these challenges, this work investigates which DL architecture can learn rich, hierarchical, and transferable representations (both fine-grained local details and global contextual patterns) from leaf images. Multiple pre-trained architectures like DenseNet121, ResNet152V2, MobileNetV2, InceptionV3, and YOLOv12 were fine-tuned and tested on the tomato and soybean leaf datasets to investigate their potential for extracting discriminative features that lead to high-accuracy disease classification. DenseNet121 with high-dense connectivity and feature reuse, ResNet152V2 with deep residual learning to ensure good gradient flow, MobileNetV2 having lightweight and resource-efficient architecture, InceptionV3 for multi-scale feature extraction, and YOLOv12 for real-time detection and localization. Additionally, a hybrid attention- based stacking ensemble model was proposed by integrating ResNet152V2 and VGG19 along with CBAM and EfficientNetB0 with a spatial attention mechanism, which augments the model’s ability to focus on disease regions over disease-infected areas and enhances precision in classification. This entire investigation facilitated the development of a high-performance, explainable, and computationally inexpensive DL framework that is not only dependable in plant disease detection but also can be effectively integrated and utilized within smart farming and precision agriculture applications.

### 2.8 Environment setup

The proposed study was performed within a conventional deep learning experimental setup in a limited resource setting, guaranteeing both model training and evaluation efficiency, as shown in **Table 3**. The experiments followed the same data preparation and training protocol, like the input size, batch processing, learning-rate choice, and dataset splitting, to estimate performances reliably. The entire procedure was a compromise between computational cost, reproducibility, and stability of the results. An early stopping was used while training to avoid overfitting and to make.

**Table 3:** Experimental setup and implementation environment.

Resource	Details
CPU	Intel^®^ Core™ i3-1005G1 @ 1.20 GHz
RAM	12 GB
GPU	NVIDIA GPU (Kaggle/Tesla)
Software/Tools	Python, TensorFlow, Keras, Jupyter Notebook
Image Size	224 × 224 × 3
Batch Size	16
Learning Rate	0.0001
Data Split	80% Training, 10% Validation, 10% Testing
Libraries	scikit-learn, TensorFlow/Keras, NumPy, Pandas, Matplotlib, Seaborn

the convergence more stable. The training status was monitored by validation loss with a patience of 10 epochs, and the best weights were automatically restored. The maximum number of epochs was set as 100 for classification models. Furthermore, a learning-rate reduction on plateau policy was used with factor 0.1 when validation performance was not improving.

### 2.9 Baseline classification models

The current section presents a brief discussion about various existing studies, like DenseNet-121, MobileNet-V2, ResNet152-V2, and Inception-V3, followed by the proposed hybrid attention model.

### 2.10 DenseNet-121

**Figure 4:**
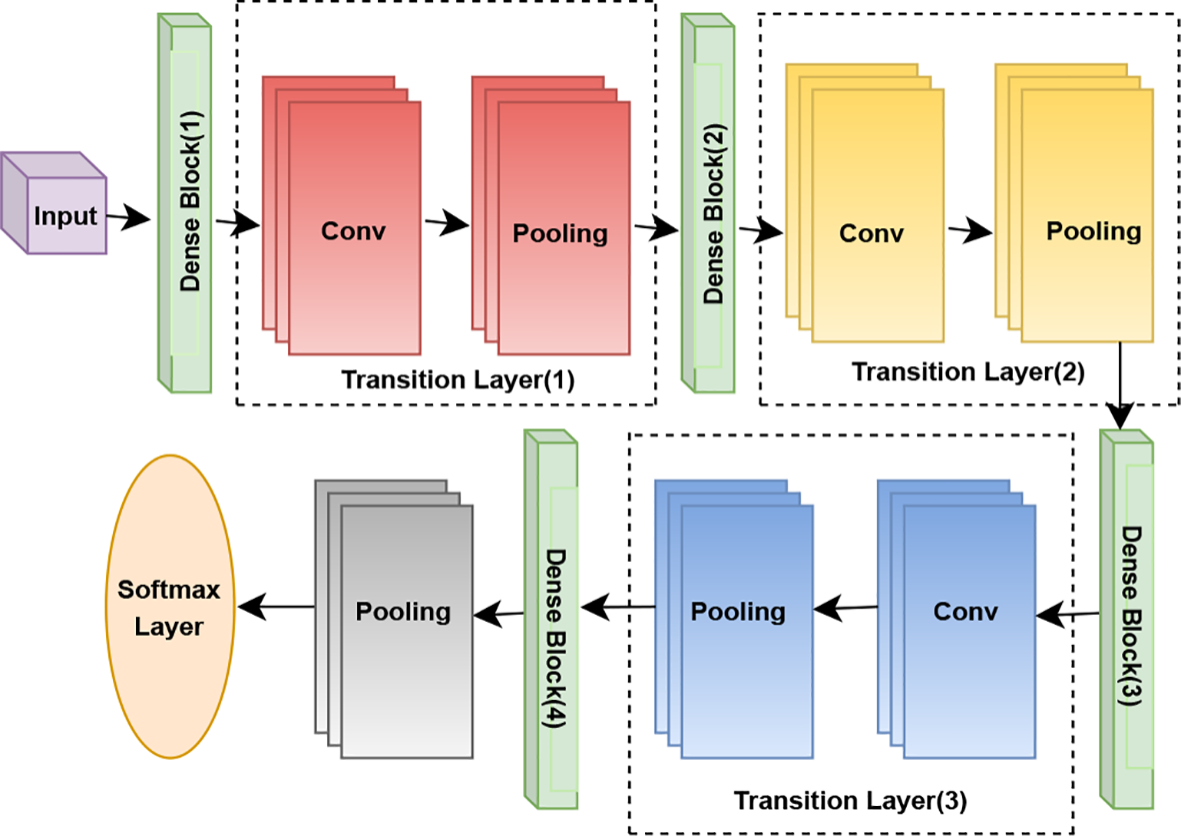
Architecture diagram of DenseNet-121.

DenseNet121 (Densely Connected Convolutional Network) (Nandhini and Ashokkumar, 2022) is a deep CNN architecture that connects each layer to every other layer in a feed-forward fashion. Rather than learning redundant features, DenseNet reuses features by concatenating feature maps learned by previous layers, which also leads to better flow of information and gradients throughout the network, as **Figure 4** shows. In this study, the model DenseNet121 (Trivedi et al., 2024) is used as the base model for feature extraction and classification to detect multiple diseases of crop leaves from the soybean dataset. The model processes preprocessed and augmented images at the input and runs them on several dense blocks where every one of these layers takes the feature maps of all previous layers as inputs. The learned features are then passed through a GAP layer, followed by fully connected layers with dropout regularization to mitigate overfitting. At last, a softmax activation function is used to classify the leaf images into the corresponding disease classes. DenseNet121 takes advantage of the pre-trained ImageNet weights and fine-tunes the weights for plant disease classification, resulting in better stability and higher accuracy in recognition work in comparison with typical CNNs. The matrix form of DenseNet121 is denoted as Equation 10.

(10) $$x_{l}=H_{l}([x_{0},x_{1},x_{2},\ldots ,x_{l-1}])$$

where $H_{l}(\cdot )$ represents a composite function of Batch Normalization (BN), Rectified Linear Unit (ReLU), and Convolution (Conv). The term $\left[x_{0},x_{1},\ldots ,x_{l-1}\right]$ denotes the concatenation of the feature maps produced by layers 0 through l − 1. This dense connectivity pattern strengthens feature propagation, encourages feature reuse, and substantially reduces the number of parameters.

### 2.11 MobileNet-V2

**Figure 5:**
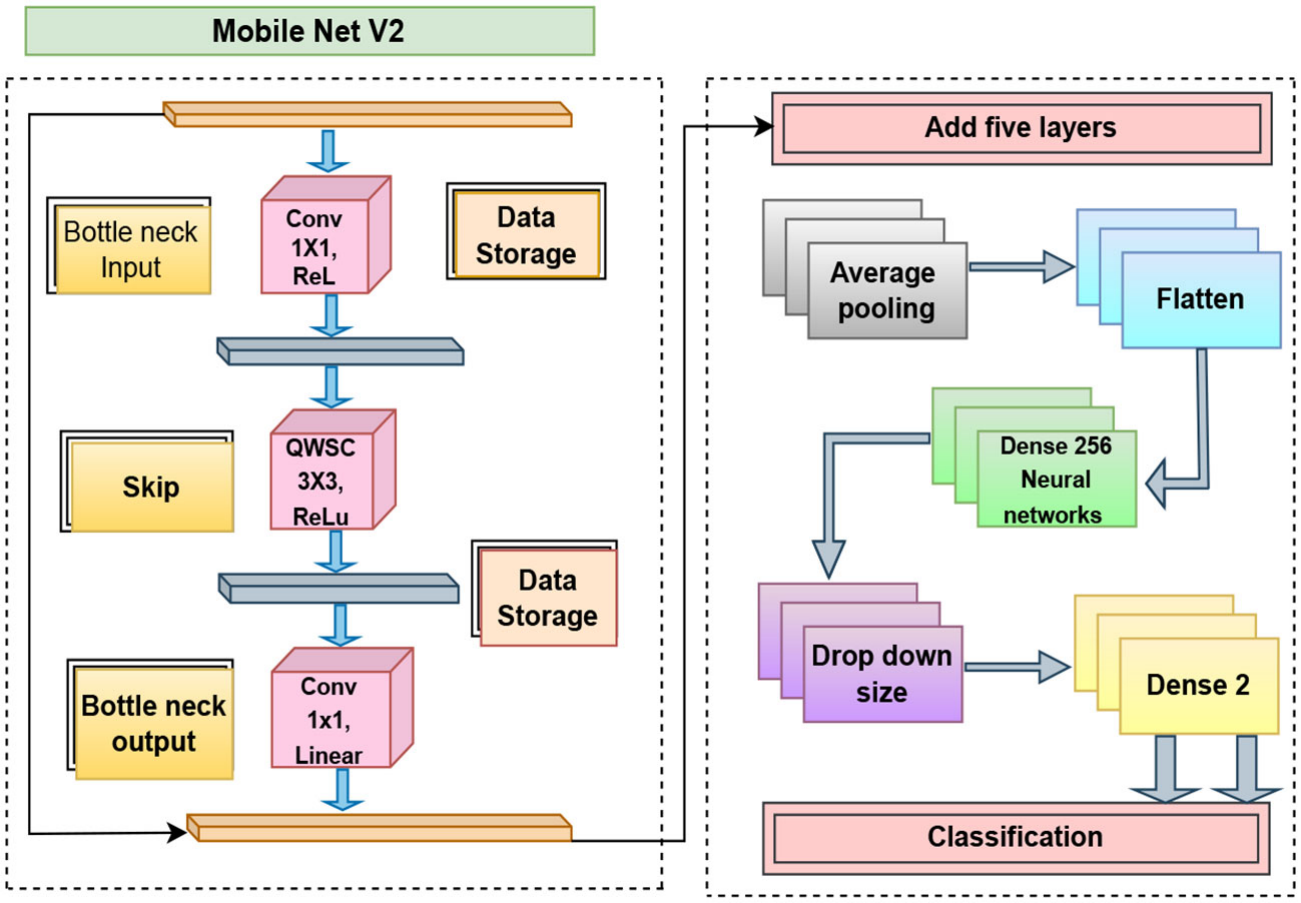
Architecture diagram of MobileNetV2.

MobileNetV2 (Zheng et al., 2025) is a simple and efficient CNN architecture tailored for mobile and resource-limited environments with high classification accuracy. The architecture is based on depthwise separable convolutions, and it hinges on two novel ideas, inverted residuals and linear bottlenecks, that allow it to significantly reduce the number of operations and the number of trainable parameters **Figure 5**. In this research work, MobileNetV2 was employed for the automatic classification of plant leaf diseases owing to its best trade-off between accuracy and inference speed. The model utilizes the pre-trained ImageNet weights for transfer learning and fine-tunes the weights on the plant disease dataset to learn distinctive features of the leaf texture, color patterns, and infected regions. The first depthwise convolution separates the spatial features, then a pointwise convolution combines them efficiently. The addition of inverted residual connections, also referred to as skip connections in residual networks ensures that information from previous layers facilitates better feature representation as well as reduces excess gradient degradation. In the end, a GAP layer and a multiple fully connected layers with a softmax activation function is used to discriminate the images to corresponding disease classes.

The standard convolution procedure, as calculated in the MobileNetV2 architecture, is defined as follows as Equation 11.

(11) y=PConv(DConv(x))

where DConv(x) denotes the *depthwise convolution* that is applied independently to each input channel and PConv(x) represents the *pointwise convolution* (a 1 × 1 convolution) that linearly combines the outputs of the depthwise convolution. Hence, the depthwise and the pointwise convolutions are executed in a sequence, which makes a depthwise separable convolution.

MobileNetV2’s architectural efficiency enables it to generalize across various disease patterns and provide robust classification performance even on large-scale agricultural image datasets. This results in an excellent trade-off among accuracy, generalization capability, and computational cost (**Figure 5**).

### 2.12 ResNet152-V2

**Figure 6:**
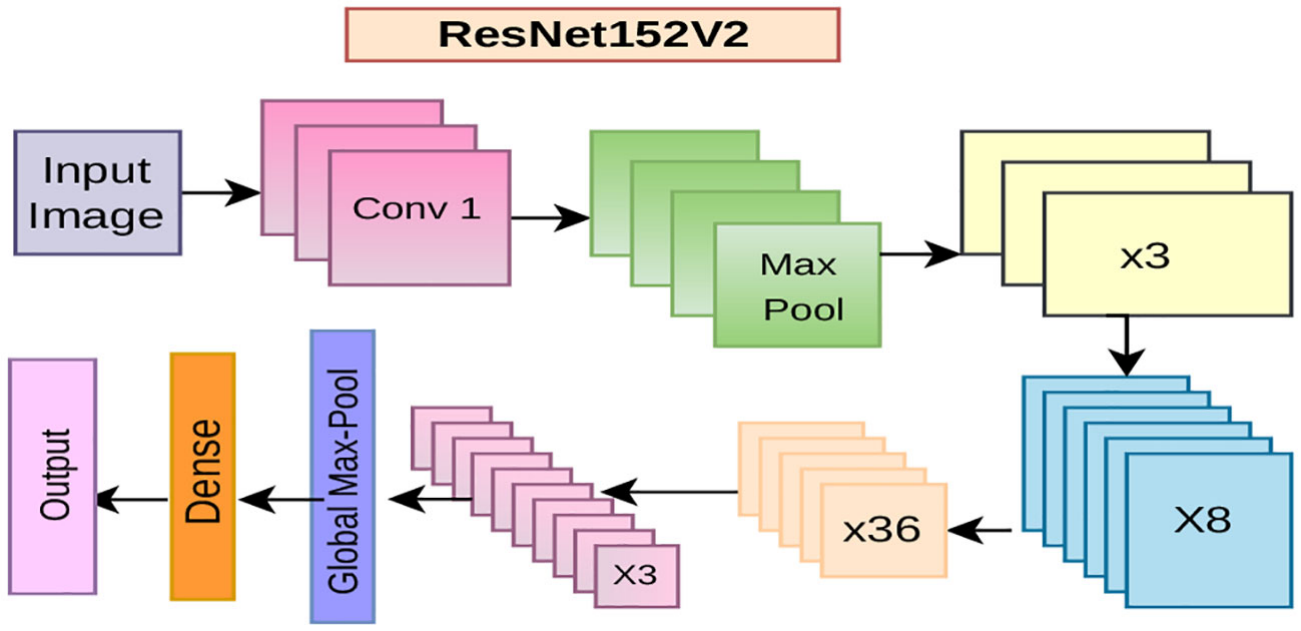
Architecture diagram of ResNet152-V2.

ResNet152V2 (Residual Network Version 2) (Gopalan et al., 2025; Padshetty and Ambika, 2023) is a modernized deep CNN architecture, which solves the vanishing gradient problem by introducing skip (residual) connections. These connections enable the gradients to flow directly, making learning more efficient and facilitating very deep architectures to be trained. Each of the 152 layers is intended to enhance feature representation by performing an element-wise addition between identity mappings and learned transformations (**Figure 6**). In this study, ResNet152V2 was fine-tuned via transfer learning with pre-trained ImageNet weights for tomato and soybean leaf disease classification. The deeper residual blocks lead to more powerful feature representation of complex leaf structures and disease patterns. The network was optimized by categorical cross-entropy loss and the Adam algorithm, gathering regularization methods as Equation 12, including early stop, learning rate decay, and class,

balance to avoid overfitting of the model and obtain the best performance.

(12) $$H(x)=F(x,\{W_{i}\})+x$$

where H(x) represents the desired underlying mapping, $F(x,\{W_{i}\})$ denotes the residual function to be learned (with $\left\{W_{i}\right\}$ being the set of learnable weights), and *x* is the input that is added directly through the skip (shortcut) connection.

This formulation allows the model to learn residual functions more effectively, facilitating faster convergence and achieving higher accuracy in image classification tasks (**Figure 6**).

### 2.13 Inception-V3

**Figure 7:**
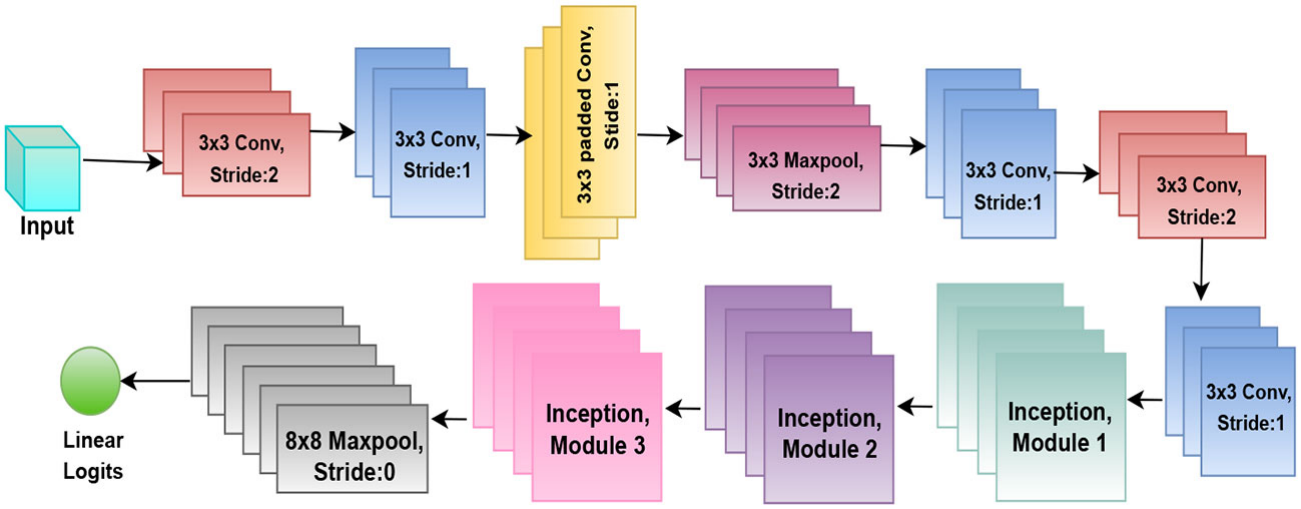
Architecture diagram of Inception-V3.

InceptionV3 (Hukkeri et al., 2024; Kumar and Kumar, 2025) is a deep CNN architecture giving an advanced feature extraction based on factorized convolutions, asymmetric filters, and multiple parallel convolutional layers within the inception module structure. Such modules allow the network to extract both high-resolution local features and low-resolution contextual features at once and have been demonstrated to be effective for challenging image classification problems. This study tunes InceptionV3 with pre-trained ImageNet weights to identify diseases of tomato leaves. The upper layer is where a GAP layer is followed by a fully connected dense layer of 1024 neurons and a softmax output layer with classes number. The model was trained with the categorical cross-entropy loss function using the Adam optimizer, and to control overfitting and to deal with class imbalance, early stopping, learning rate reduction on plateau, and class weighting have been applied. In mathematical terms, the Inception module can be treated as a parallel concatenation of convolutions as Equation 13, **Figure 7**.

(13) $$y=f_{1\times 1}(x)⊕f_{3\times 3}(f_{1\times 1}(x))⊕f_{3\times 3}(f_{3\times 3}(f_{1\times 1}(x)))⊕f_{1\times 1}(\text{Pool}(x))$$

where $f_{n\times n}(x)$ is an *𝑛* × *𝑛* convolution on input *𝑥*, Pool(*𝑥*) represents a pooling operation, and ⊕ denotes concatenation along the channel dimension. This simple design enables the network to capture and operate on features at multiple scales at the same time, which significantly boosts the model’s ability to classify the disease patterns of plant leaves more precisely.

### 2.14 Hybrid attention-based ensemble model

In this paper, leaf disease classification based on a hybrid attention-based ensemble model, which includes ResNet152V2, VGG19 with CBAM, and EfficientNetB0 with spatial attention, is proposed. This multilevel representation learning framework is constructed to learn fine-grained leaf features with the most informative spatial and channel-wise features in the end. The proposed framework has three core modules: feature extraction, attention enhancement, and soft voting-based ensemble classification **Figure 25**. The input images are scaled and cropped to the same size for all three backbone networks, that is, 224 × 224 with three RGB channels. Data augmentations such as rotations, zooming, and horizontal flipping are used to enhance the generalization of the model.

The hybrid ensemble carries out probability-level fusion using equal-weight soft voting. Each backbone applies softmax activation to obtain class probabilities separately, and the final prediction is then calculated by averaging the probability vectors from ResNet152V2, VGG19+CBAM, and EfficientNetB0. The ensemble-based approach does not rely on any extra meta-learner.

The input to each of the backbone networks is an image of size 224 × 224 × 3. ResNet152V2 does feature extraction and global average pooling and has a dense layer with 256 neurons. VGG19 is modified by adding a Convolutional Block Attention Module (CBAM) after the last convolution block and followed by global average pooling and a fully connected layer of 256 units. A spatial attention module is also embedded in EfficientNetB0 before pooling. Every backbone representation is converted into a probability vector via softmax activation.

The whole pipeline of the proposed hybrid model is as follows: (1) preprocessing and augmentation; (2) multi-branch feature extraction based on ResNet152V2, VGG19+CBAM, EfficientNetB0 with spatial attention; (3) single classification by FC layers and softmax outputs for each branch; and (4) soft-voting fusion at the probability level to obtain the final prediction of the disease. This strategy guarantees that complementary spatial and channel-wise attention features are integrated while maximizing the architectural independence among backbones.

### 2.15 YOLOv12

YOLOv12 is a new single-stage object detection framework that can achieve the high accuracy of two-stage methods while ensuring good real-time inference speed. Different from classification-based CNNs, YOLOv12 treats disease identification as a single regression problem and predicts the bounding box coordinates along with the object confidence scores and the class probabilities in one single forward pass.

The detection task focuses on localizing symptomatic leaf regions. The bounding box annotations represent a new contribution in this work and cover the whole disease area of a leaf, as opposed to using existing tags obtained from publicly available datasets that typically lack spatial coordinate annotations. A macro-level detection strategy was adopted instead of detecting individual lesions. So, the framework would best serve real-time precision agriculture, as knowing the existence and location of a diseased leaf is more actionable information for site-specific treatment than that of single lesions.

For high-fidelity ground truth, a group of three annotators, all with a background in agricultural science, manually annotated all images using the LabelImg graphical interface tool. The annotation scheme adhered to rigorous rules: the boxes have to stipulate a two-pixel boundary around visible chlorotic or necrotic margins and should exclude any non-leaf background contaminants. The following three-stage quality control protocol was applied: (1) independent annotation of the dataset, (2) a cross-check phase during which a second annotator evaluated the tightness of the boxes and the accuracy of the assigned class of a 100% portion of the dataset, and (3) a final arbitration phase by a senior researcher to settle any classification conflicts. This rigorous procedure minimizes labeling bias and guarantees the spatial accuracy necessary for robust model convergence.

The YOLOv12 framework, as shown in **Figure 26**, is composed of three stages: a backbone network that exploits the hierarchical features, a neck component that performs multi-scale feature fusion, and a dense prediction detection head. To provide a robust model for subsequent learning, the backbone is designed to capture both low-level textures and high-level semantics of leaves, which are critical for learning fine-grained disease signs, for example, spots, discolorations, and lesion shapes. The neck utilizes path enhancement aggregation to combine feature maps at different spatial scales for effective detection of disease regions with sizes, shapes, and orientation variability.

YOLOv12 formulates object detection as a single regression problem in a mathematical sense. The model also predicts the center coordinates for each bounding box as (x, y), width *w*, height *ℎ*, objectness confidence $P_{obj}$, and class probability $P_{cls}$. The overall training loss function is defined as shown in Equation 14:

(14) $$L_{YOLO}=L_{bbox}+L_{obj}+L_{cls}$$

where $L_{bbox}$ represents the bounding box regression loss based on Intersection over Union (IoU), $L_{obj}$ denotes the objectness loss, and $L_{cls}$ is the classification loss computed using categorical cross-entropy. In this study, the YOLOv12n model from the Ultralytics framework was adopted as the detection backbone (Ultralytics, 2024). We did not make any architectural changes; the detector was used as provided to ensure a fair comparison with existing object detection frameworks. The training and augmentation parameters were the conventional ones with task-specific tuning for agricultural leaf images.

### 2.16 Performance strategies

**Figure 8:**
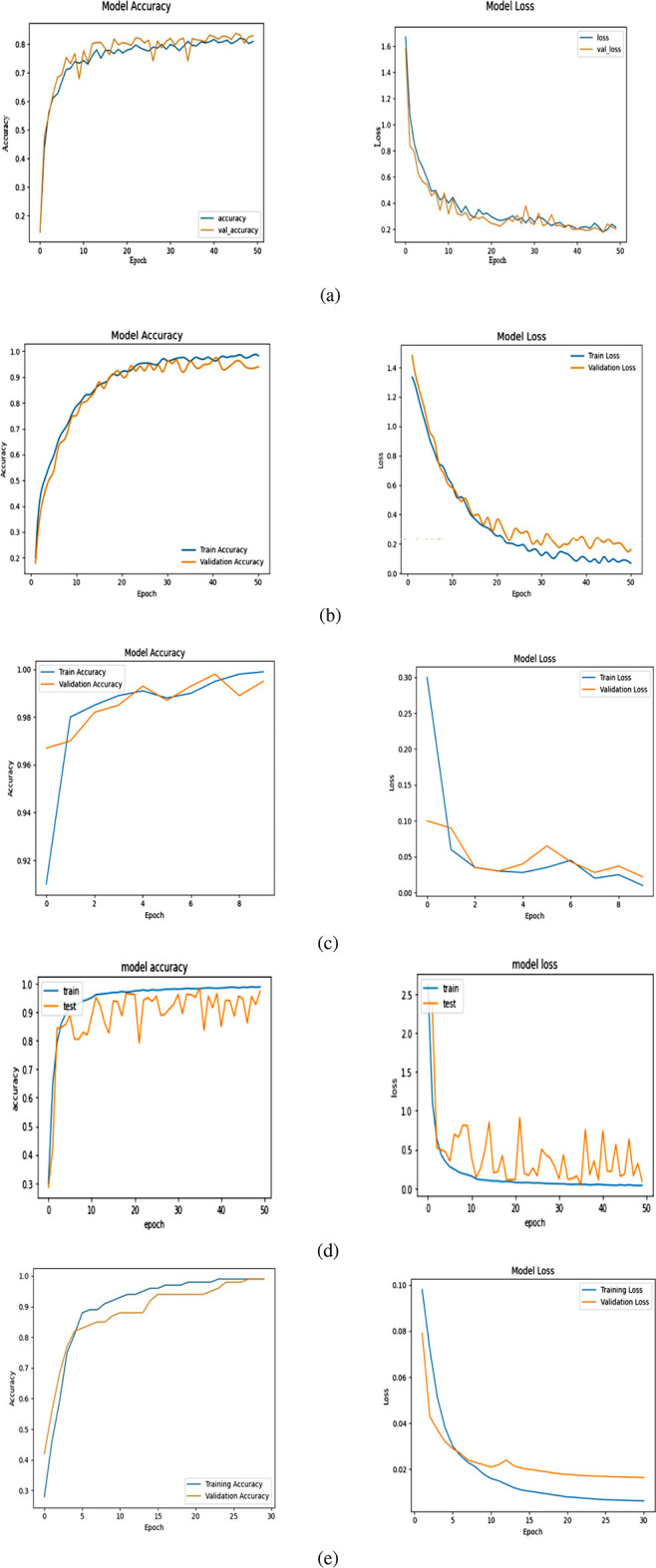
Tomato leaf dataset results: **(a)** MobileNetV2, **(b)** ResNet152V2, **(c)** InceptionV3, **(d)** DenseNet121, and **(e)** Hybrid model, showing training accuracy and loss curves.

**Figure 9:**
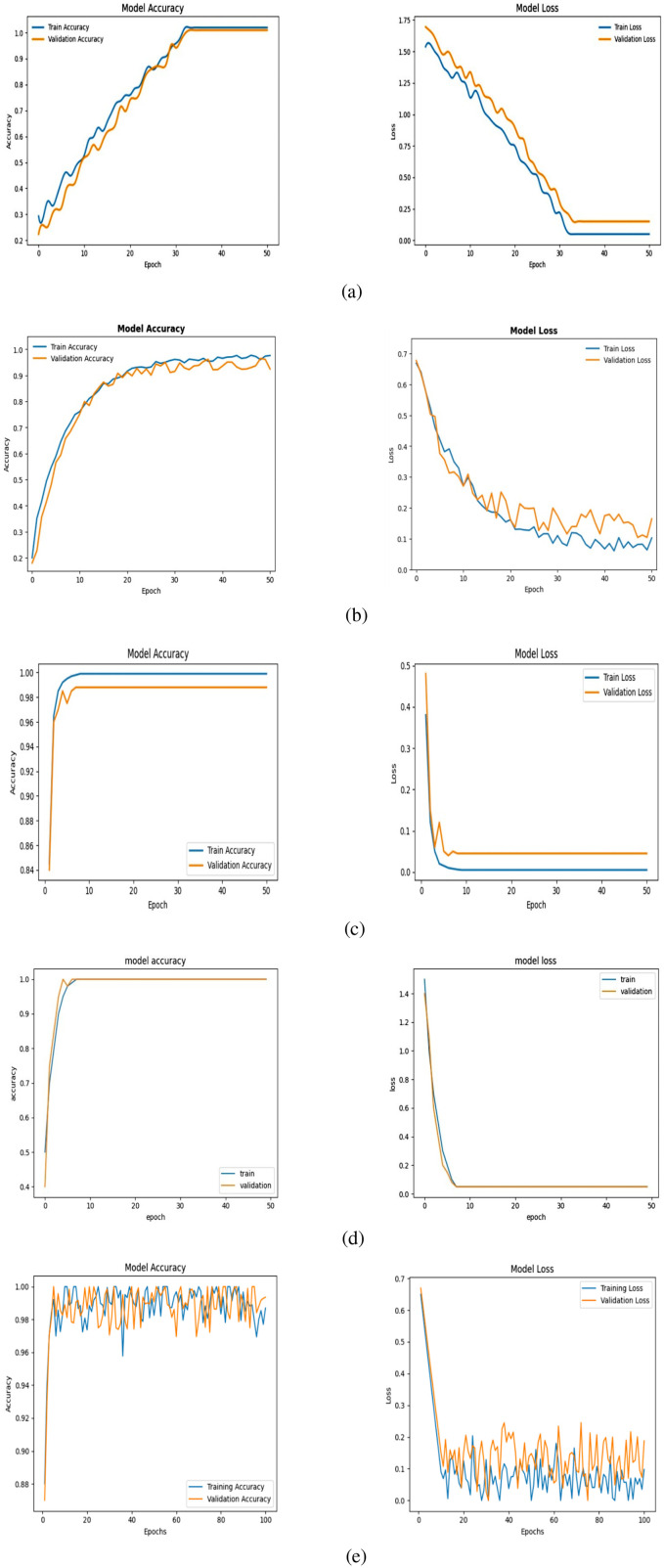
Soybean leaf dataset results: **(a)** MobileNetV2, **(b)** ResNet152V2, **(c)** InceptionV3, **(d)** DenseNet121, and **(e)** Hybrid model, showing training accuracy and loss curves.

This section shows a full performance evaluation of our proposed deep learning-based models with the training and validation accuracy/loss curves shown in **Figures 8**, **9**, as well as the standard evaluation metrics. The aim of this analysis is to examine model convergence, learning stability, and generalization for a variety of different model architectures and datasets.

Model accuracy and model loss, on the other hand, are presented in **Figures 8**, **9** for a number of popular deep learning architectures, such as MobileNetV2, ResNet152V2, InceptionV3, DenseNet121, and the proposed hybrid model, evaluated on the Tomato Leaf Dataset and the Soybean Leaf Dataset in that order. Accuracy of the model is the number of correctly classified samples, while the model loss (categorical cross-entropy) is the measure of prediction error during optimization.

In all models, a sharp rise in the training as well as validation accuracy during the first epochs is clearly visible, suggesting that features were extracted successfully and the discriminative features have been learned. During training, the accuracy curves become increasingly smooth as the curves converge to the approximate optimal model parameters. The training and validation accuracy curves are close in most of the results, which indicates that the models generalize well for unseen data and there is minimal overfitting. Small fluctuations in validation accuracy, particularly for deeper architectures, are attributed to batch-wise variations and dataset complexity.

**Figure 8** verifies that the proposed hybrid model achieves better validation accuracy and loss with fewer epochs on the tomato leaf dataset as compared to the other architectures. **Figure 9** shows a similar phenomenon for the soybean leaf dataset, demonstrating that the proposed method achieves stabler results across the tested crop datasets. However, to fully generalize to other crops, imaging devices, and field conditions, further testing on more diverse datasets is needed. These results indicate that the hybrid structure is better equipped to handle both inter-class similarity and dataset variation. In summary, the reporting strategies used in this paper, including monitoring trends of accuracy loss, validating the stability of convergence, and assessing various complementary metrics, provide evidence that the proposed models, especially the hybrid architecture, enable efficient learning, promising generalization, and reliable disease classification results.

## 3 Results

The proposed framework has been trained and tested on the dataset using an 80:10:10 ratio for training, validation, and testing with stratified sampling while preserving the original class distribution of the tomato and soybean datasets using stratified sampling without performing any artificial class balancing.

While several evaluation metrics are provided to provide a comprehensive assessment of the performance, the main metrics that are considered for model comparison in this work are the accuracy, F1-score, balanced accuracy, and mAP (for detection). Among these, accuracy offers an overall measure of correctness; the F1-score balances precision and recall in case of class imbalance; balanced accuracy accounts for biased class distributions; and mAP is the standard performance metric for object detection. Other so-called “reported metrics,” like MCC, Cohen’s Kappa, log loss, Hamming loss, F2-score, and the Jaccard index, are presented as additional indicators for validating the robustness.

The selection of several evaluation criteria is motivated by the need to evaluate the model stability from different statistical points of view. Although accuracy represents the entire classification result, metrics including MCC and Cohen’s Kappa make use of true and false predictions of all classes and are less influenced by class imbalance. Balanced accuracy is especially important to be considered in agricultural datasets where some classes of diseases may contain fewer numbers of samples. It uses an attention-guided feature extraction mechanism to learn both fine-grained and high-level leaf features, thus facilitating the discrimination capabilities of the model. We evaluated the system performance with several well-known evaluation metrics, namely, accuracy, precision, recall, F1-score, and balanced accuracy, to quantify how well the proposed system predicts. We also used the confusion matrix to visualize the classification results in different classes, as it highlights how well the model can correctly recognize the disease patterns with less misclassification. The results confirm that the suggested system can obtain reliable classification performance and efficiently deal with the within-class variation to enhance the prediction performance. The strong performance across models is to be expected within controlled benchmark settings, and further discussion regarding dataset properties and leakage prevention methods can be found in Section 4.

Confidence intervals are not explicitly reported, but the deterministic experimental setup and similar validation performance across epochs suggest stable training of the model. Future work may consider cross-validation or multi-seed evaluation to additionally measure the variation across random initializations.

### 3.1 Confusion matrix

**Figure 10:**
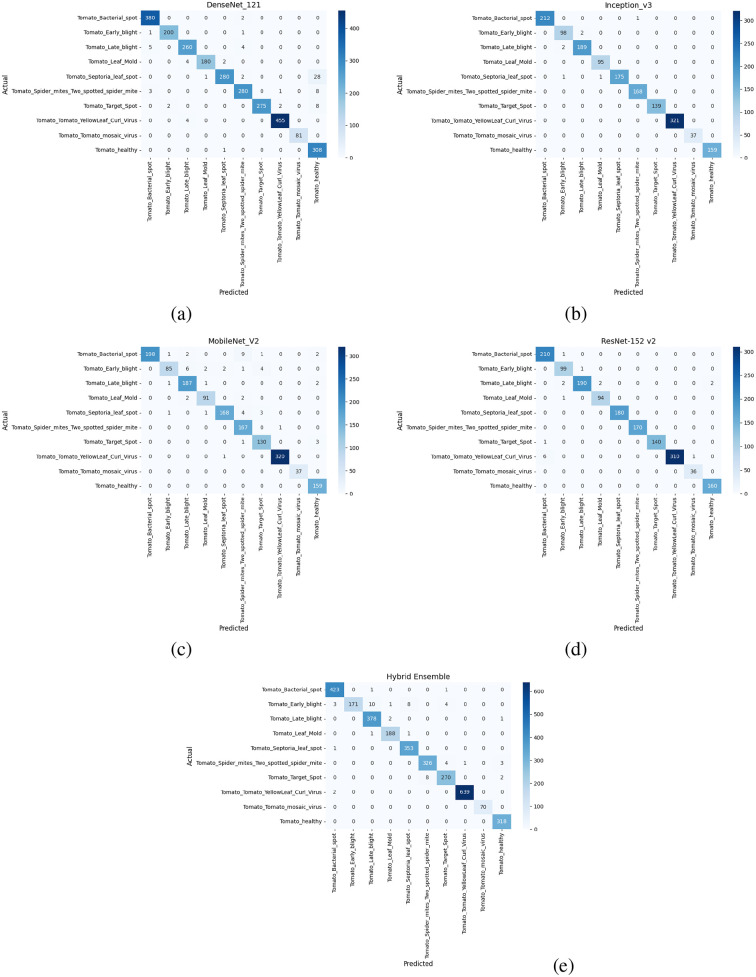
Confusion matrix for tomato leaf dataset **(a)** DenseNet-121 **(b)** Inception-V3 **(c)** MobileNet-V2 **(d)** ResNet152-v2 **(e)** Hybrid Ensemble.

**Figure 11:**
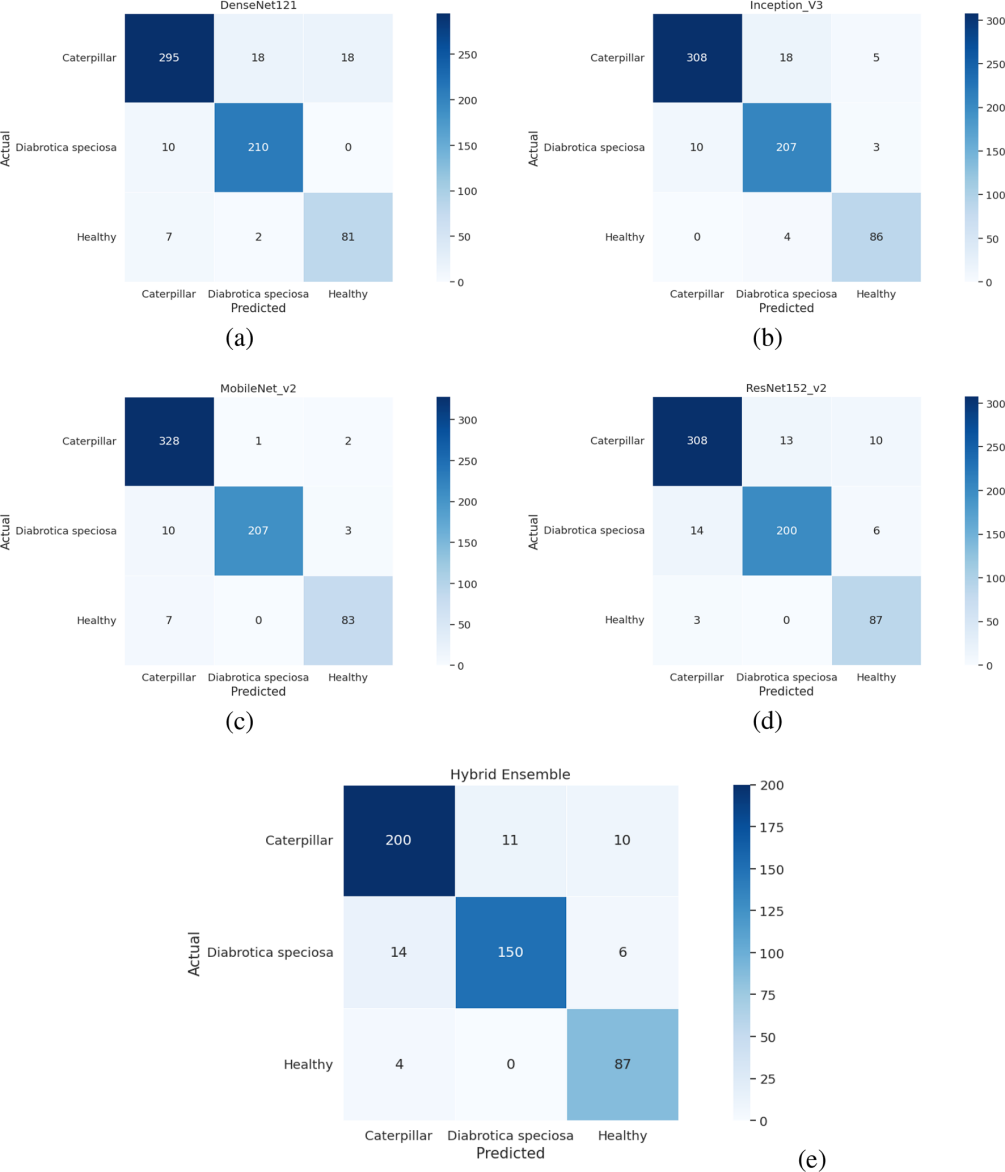
Confusion matrix for soybean leaf dataset **(a)** DenseNet-121 **(b)** Inception-V3 **(c)** MobileNet-V2 **(d)** ResNet152-v2 **(e)** Hybrid Ensemble.

The tomato and soybean leaf confusion matrices, **Figures 10**, **11**, also depict the situation that the classification models obtain good predictive performance on all disease categories. Confusion matrices show strong class-wise prediction accuracy with minimal misclassification between visually similar disease classes. These models successfully discriminate reliably between diseases that have markedly different patterns of symptoms, and the small amount of misclassification observed tends to occur between those classes that have shared symptoms (e.g., similar lesion shape, color patterns, and extent of chlorosis), and this is expected as these shared symptoms naturally increase the inter-class ambiguity. However, the relatively low error rates on both the datasets illustrate the robustness of the models with respect to changes in illumination conditions, leaf orientations, background texture, symptom severity, and so forth on the leaves. Taken together, these results demonstrate that the proposed classification method is well generalized to different plant species, and it can discriminate a diverse set of disease classes with high accuracy, thereby addressing the challenge for practical use in automated plant health monitoring and diagnostics in agriculture. In addition, the similar patterns across the datasets are indicative of the model’s strong cross-domain adaptability. High per-class recognition rates further confirm its applicability to field crop surveillance. In a nutshell, the results of confusion matrices justify the robustness and applicability of the presented disease classification technique.

All confusion matrices are an exportable high-resolution file with standardized font sizes and clear labeling of your classes to make sure they are readable at scale for publication.

As shown in **Table 4**, the Grad-CAM–based XAI results highlight disease-specific regions such as lesions, necrotic areas, mosaic patterns, and pest-affected zones, while healthy leaves exhibit more uniform activations. This confirms that the model relies on biologically meaningful features, thereby enhancing interpretability and trust in tomato disease diagnosis Samek et al. (2017).

**Table 4:** Grad-CAM visualizations showing the discriminative regions used by the model for classifying different tomato leaf disease categories.

Class	Visualization
Late Blight	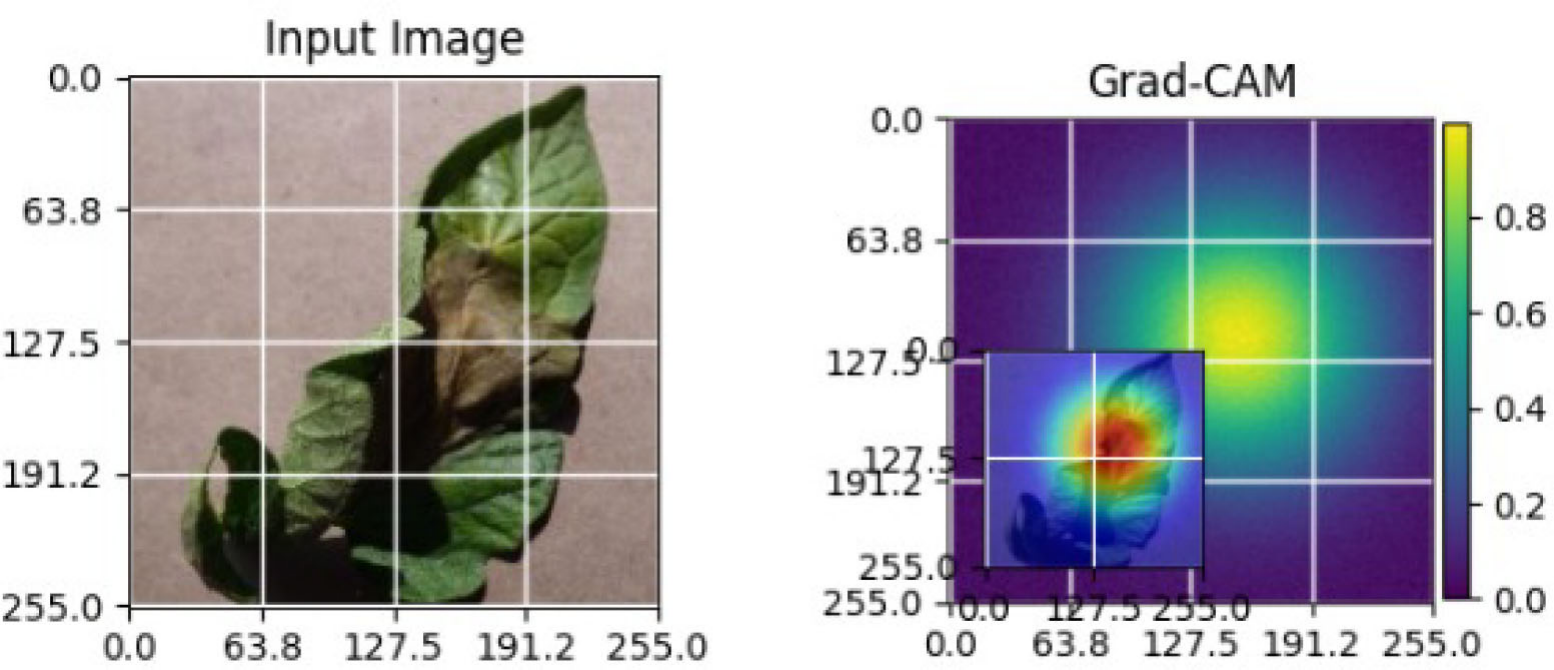
Tomato Yellow Leaf Curl Virus	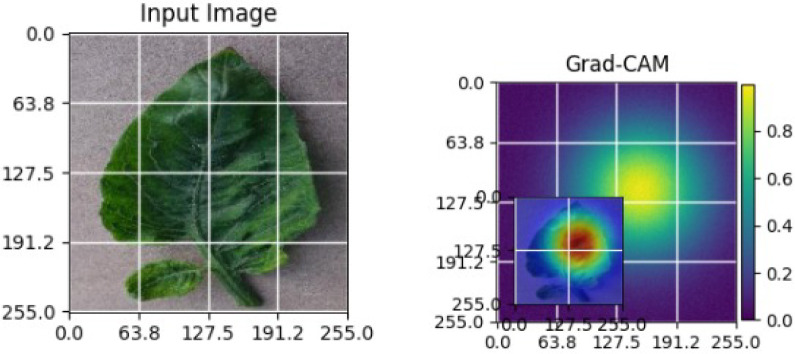
Separia Leaf Spot	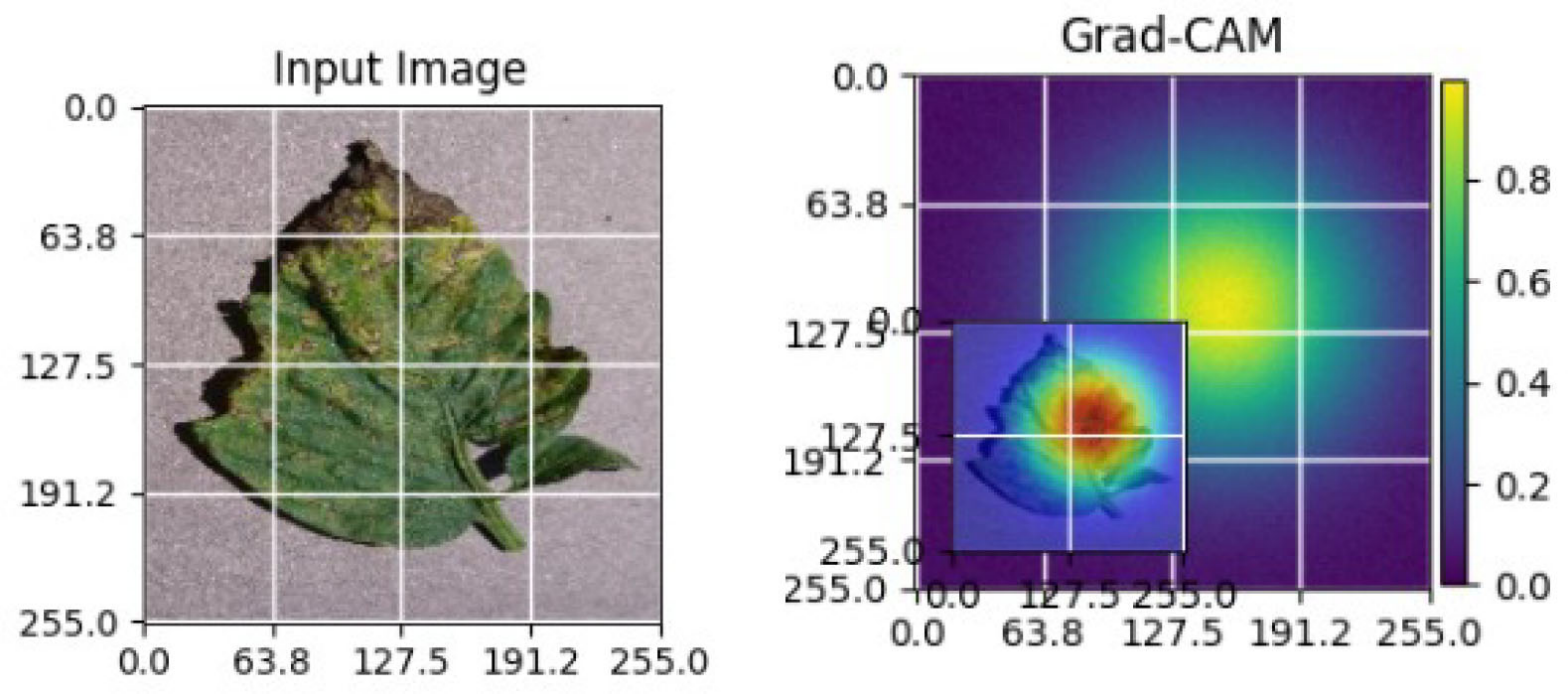
Early blight	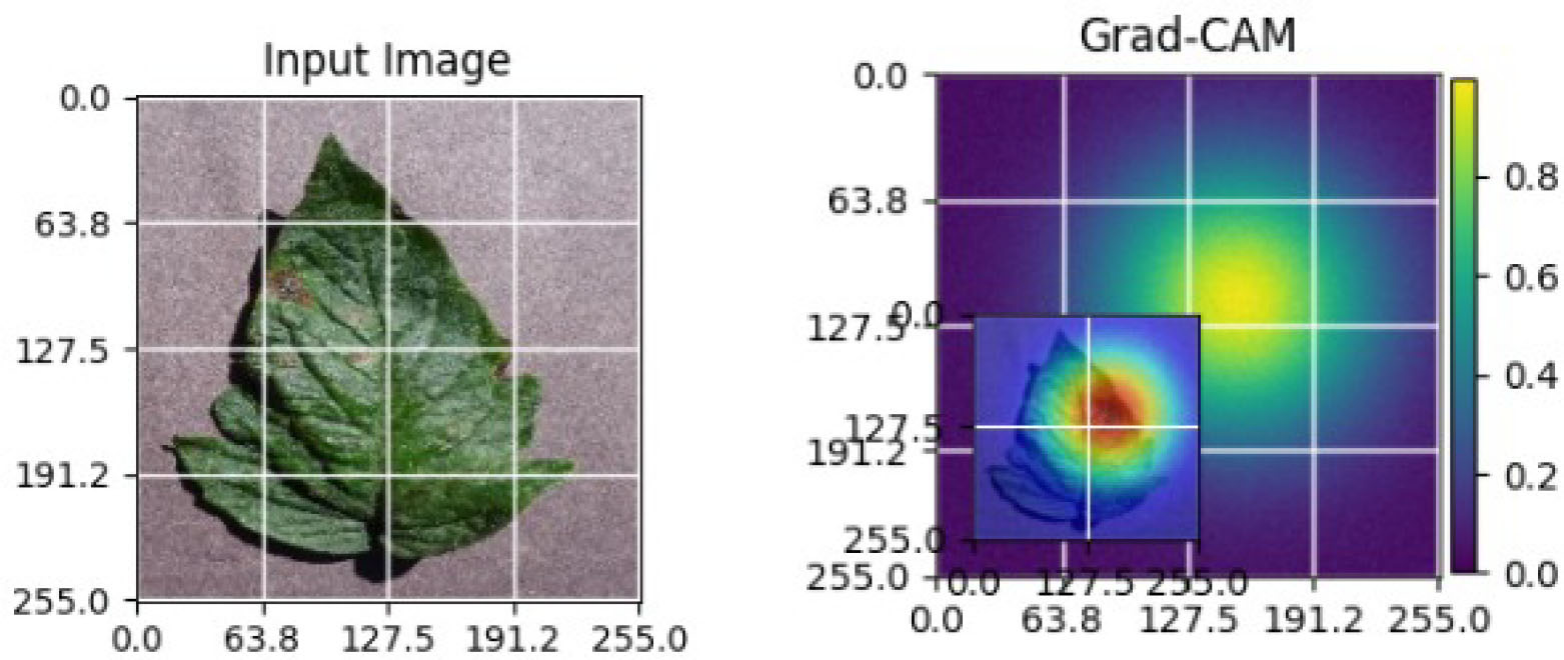
Spider Mites	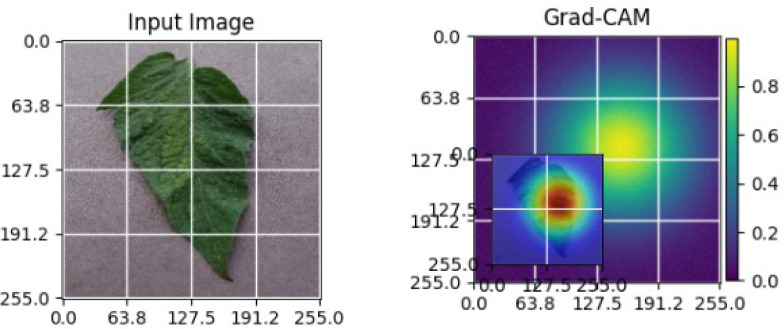
Healthy	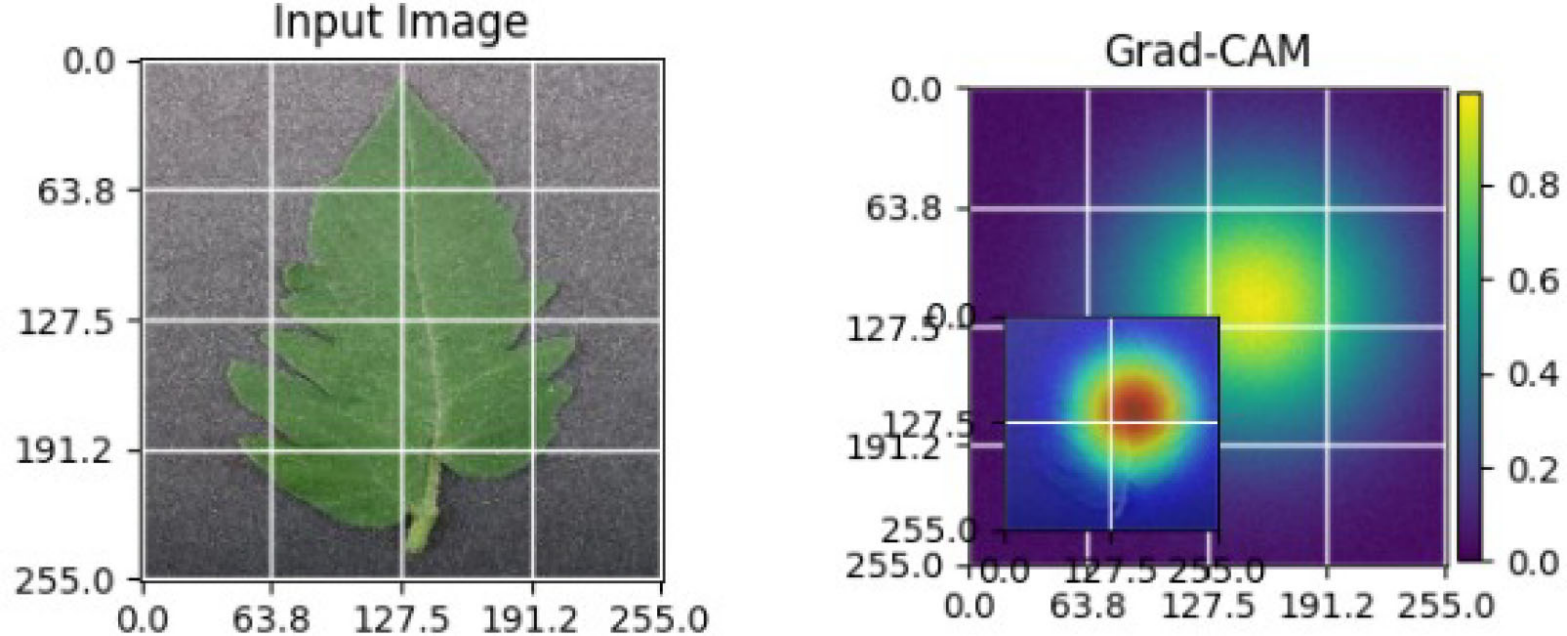
Bacterial Spot	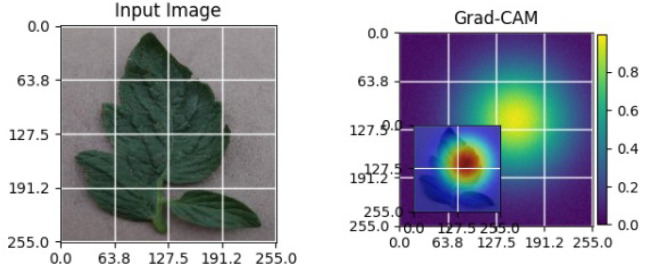
Target Spot	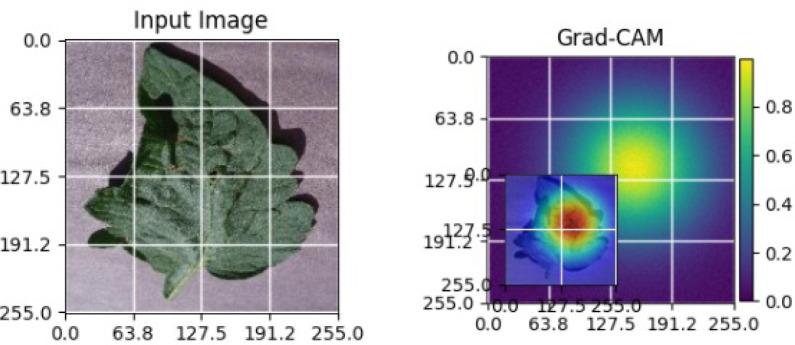
Tomato Mosaic Virus	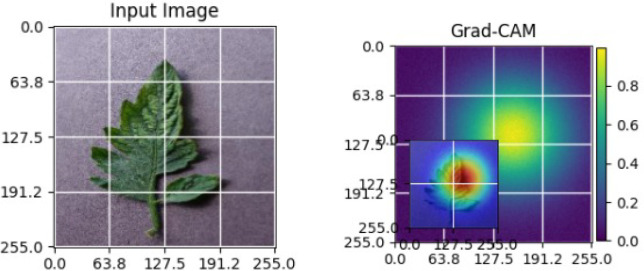
Leaf Mold	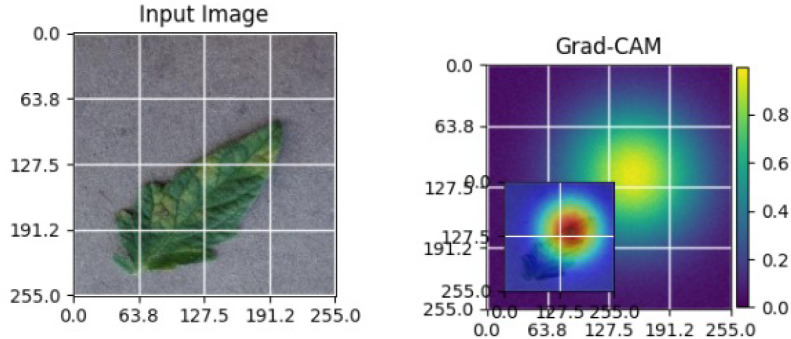

As shown in **Table 5**, the Grad-CAM activations focus on stress-related regions in diseased soybean leaves, while healthy samples exhibit diffused responses consistent with normal leaf structures. These observations demonstrate the robustness and generalization capability of the proposed XAI approach across different crops Kamilaris and Prenafeta-Boldú (2018).

**Table 5:** Grad-CAM visualizations for different soybean leaf disease categories.

Class	Visualization
Caterpillar	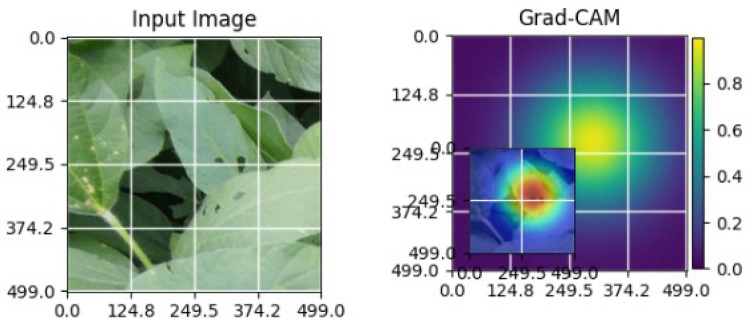
Diabrotica speciosa	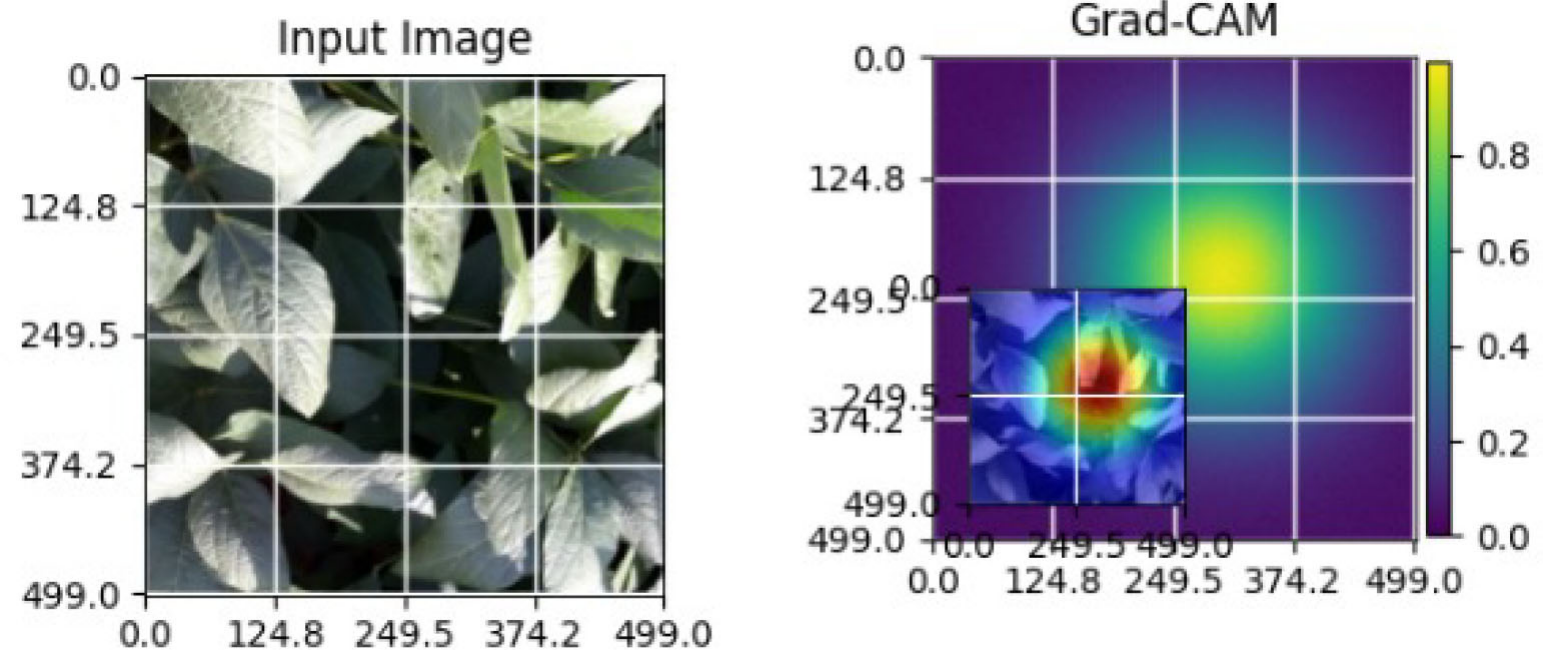
Healthy	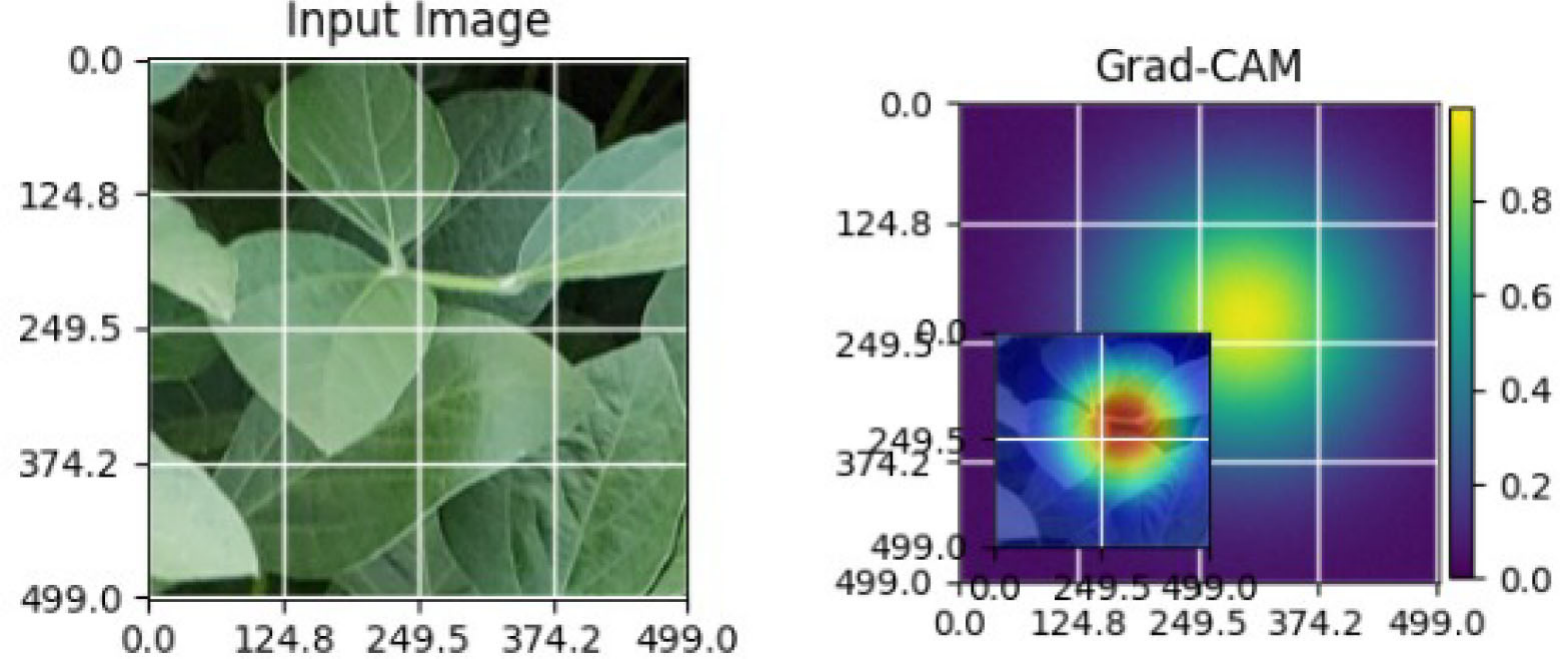

The Grad-CAM heatmaps are computed from the last convolution layer of the corresponding backbone networks, such as ResNet152V2, VGG19+CBAM, EfficientNetB0 in the proposed hybrid ensemble framework. The visualized Grad-CAMs correspond to true samples, unless otherwise stated, and we also show some failure cases for this and other tasks, when highlight model limitations is the best way to do this functionally.

The visualizations suggest that the model is primarily observing the diseased areas of the leaf as opposed to the background noise and artifacts, confirming the correctness of the learned feature representations.

### 3.2 Experimental results and performance evaluation of the YOLOv12 model

In this section, the extensive results of YOLOv12 (Guo et al., 2025) on the two standard agricultural datasets (the tomato leaf disease dataset and the soybean leaf disease dataset). The model performance is analyzed by the common evaluation indicators such as the training and validation loss curves, precision, recall, mean average precision (mAP), and confusion matrices to test the robustness of classification and generalization of the models.

#### 3.2.1 Performance evaluation on the tomato leaf disease dataset

This section describes the full results of the YOLOv12 model on the tomato leaf disease dataset. The evaluation provides training and validation learning curves, confusion matrix analysis, qualitative detection results and per-class performance measures to analyze the detection accuracy, class-wise discrimination, and overall generalization performance.

**Figure 12:**
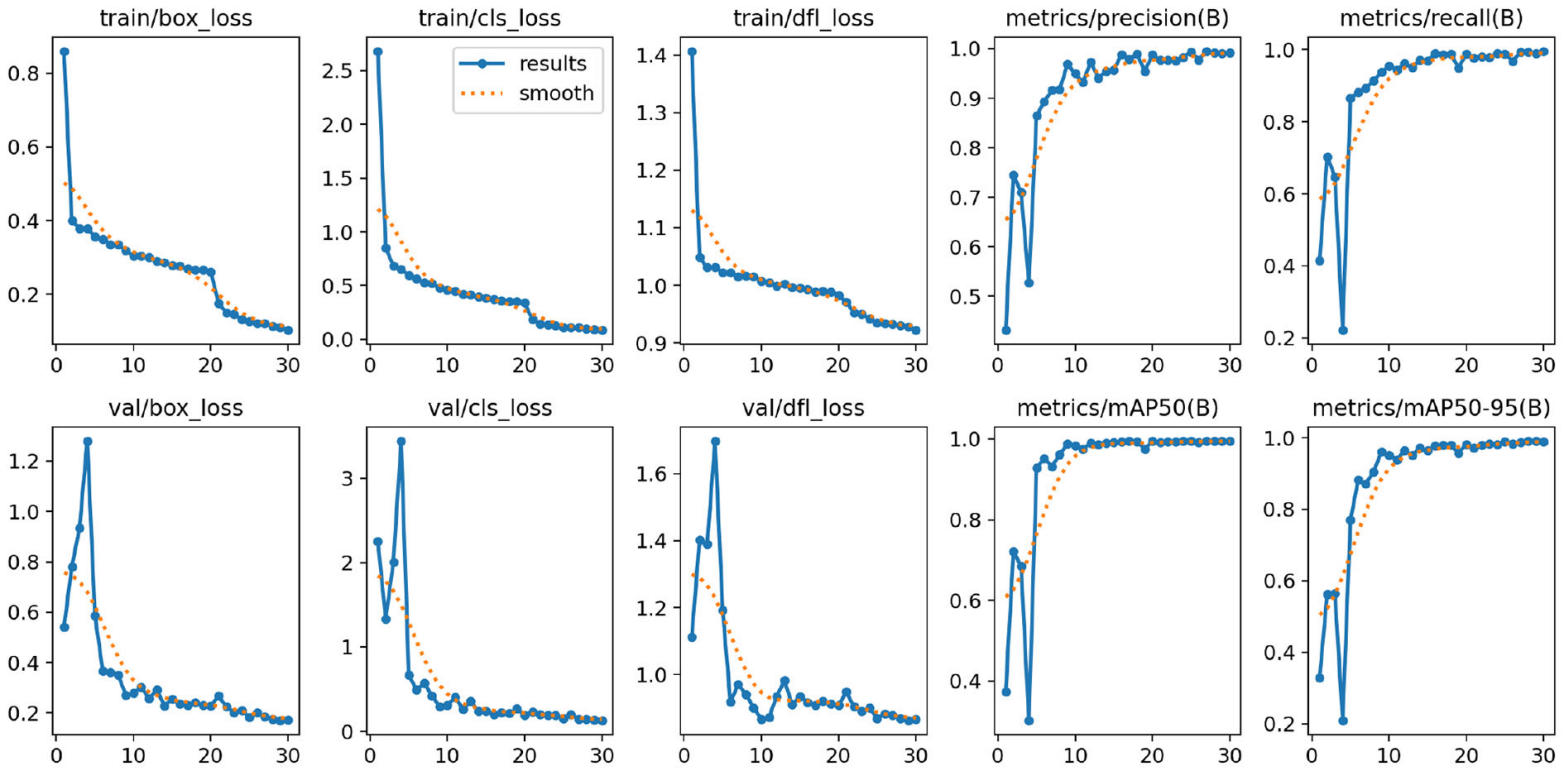
Training loss and accuracy curves of the proposed YOLOv12 model.

**Figure 12** shows the loss and accuracy curves for training the YOLOv12 model. The bounding box loss, objectness loss, and classification loss were gradually decreased, and precision, recall, and mAP were gradually increased in the whole training process. The model was found to be converged after about 95 training epochs, which also demonstrated that stable optimization was achieved with no evident overfitting or underfitting. These learning dynamics suggest that YOLOv12 generalizes well over diverse disease types and heterogeneous background conditions.

**Figure 13:**
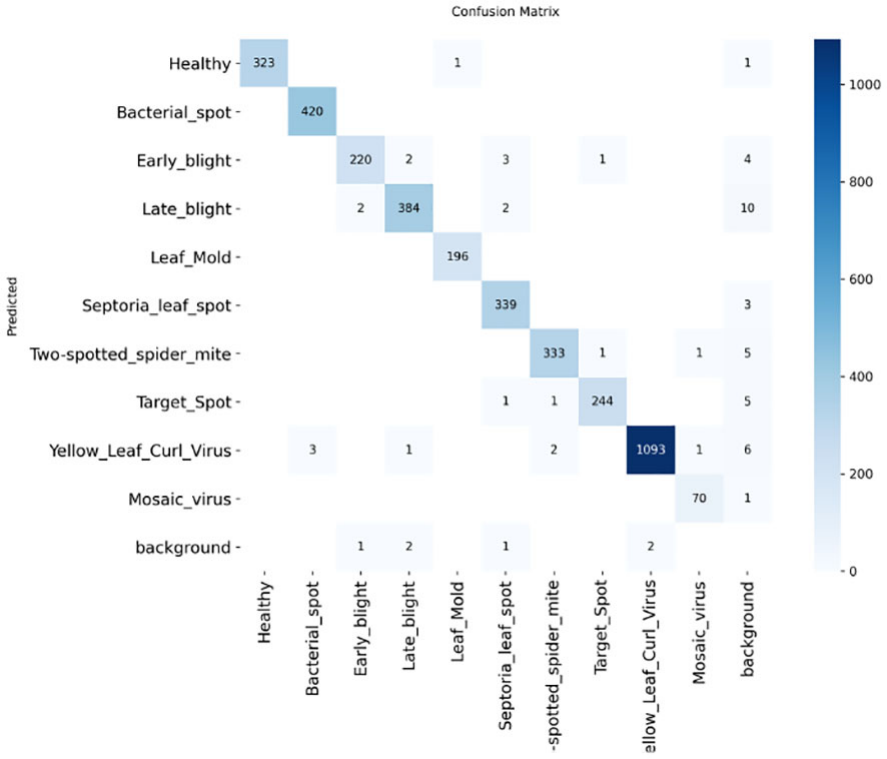
Confusion matrix of the YOLOv12 model on the test dataset.

**Figure 14:**
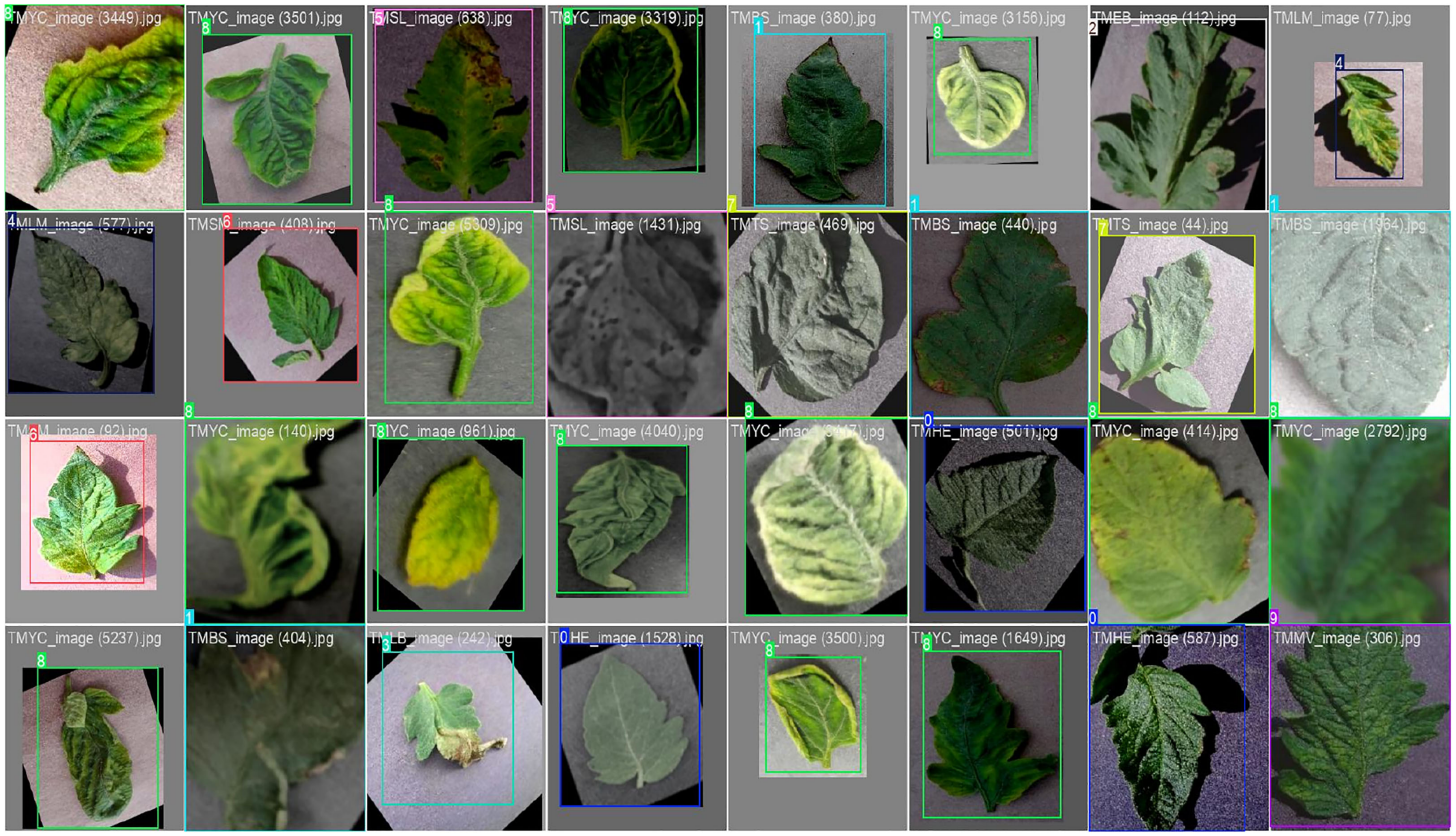
Qualitative detection results of the YOLOv12 model on the tomato leaf disease dataset. The images illustrate bounding-box localization of symptomatic tomato leaf regions across diverse backgrounds and lighting conditions.

In **Figure 1**, reffig: **Figure 13** the confusion matrix of the YOLOv12 model is shown. The values on the diagonal show that the majority of the tomato disease classes have been correctly identified, indicating high class-wise accuracy. Misclassifications were also limited and occurring mostly among visually similar disease patterns like Target spot and bacterial spot. These findings validate that YOLOv12 attains strong class separation, even in the presence of heterogeneous leaf shape and environmental conditions. The good detection results of tomato leaf diseases in qualities verify the.

effect of the YOLOv12 model in accurately detecting tomato leaf diseases in different situations. As illustrated in **Figure 14**, the proposed model can accurately localize the disease regions in the presence of illumination variation, leaf orientation, color distortion, and complicated background with tight bounding boxes and right class labels (0–9 as corresponding to healthy, bacterial spot, early blight, late blight, leaf mold, septoria leaf spot, two-spotted spider mite, target spot, yellow.

leaf curl virus, and mosaic virus). In addition, YOLOv12 has excellent discriminative power on the visually similar disease classes, which is very helpful to reduce the misclassification errors as in the case of traditional CNN-based classifiers, as shown in **Table 6**. These qualitative results are consistent with the quantitative improvements reported in the confusion matrix and further demonstrate that the method performs well for different severities of diseases, textures of leaves, and ambient environments.

**Table 6:** Per-class performance metrics of the YOLOv12 model in terms of precision, recall, mAP@0.5, and mAP@0.5:0.95.

Class	Precision	Recall	mAP@0.5	mAP@0.5:0.95
Healthy	1.0000	0.9935	0.9950	0.9950
Bacterial Spot	0.9953	1.0000	0.9950	0.9949
Early Blight	0.9667	0.9886	0.9923	0.9903
Late Blight	0.9788	0.9840	0.9909	0.9897
Leaf Mold	1.0000	0.9895	0.9941	0.9886
Septoria Leaf Spot	0.9941	0.9545	0.9766	0.9709
Two-spotted Spider Mite	0.9938	0.9877	0.9990	0.9912
Target Spot	0.9774	1.0000	0.9949	0.9912
Yellow Leaf Curl Virus	0.9945	1.0000	0.9950	0.9925
Mosaic Virus	1.0000	1.0000	0.9950	0.9950

**Figure 15:**
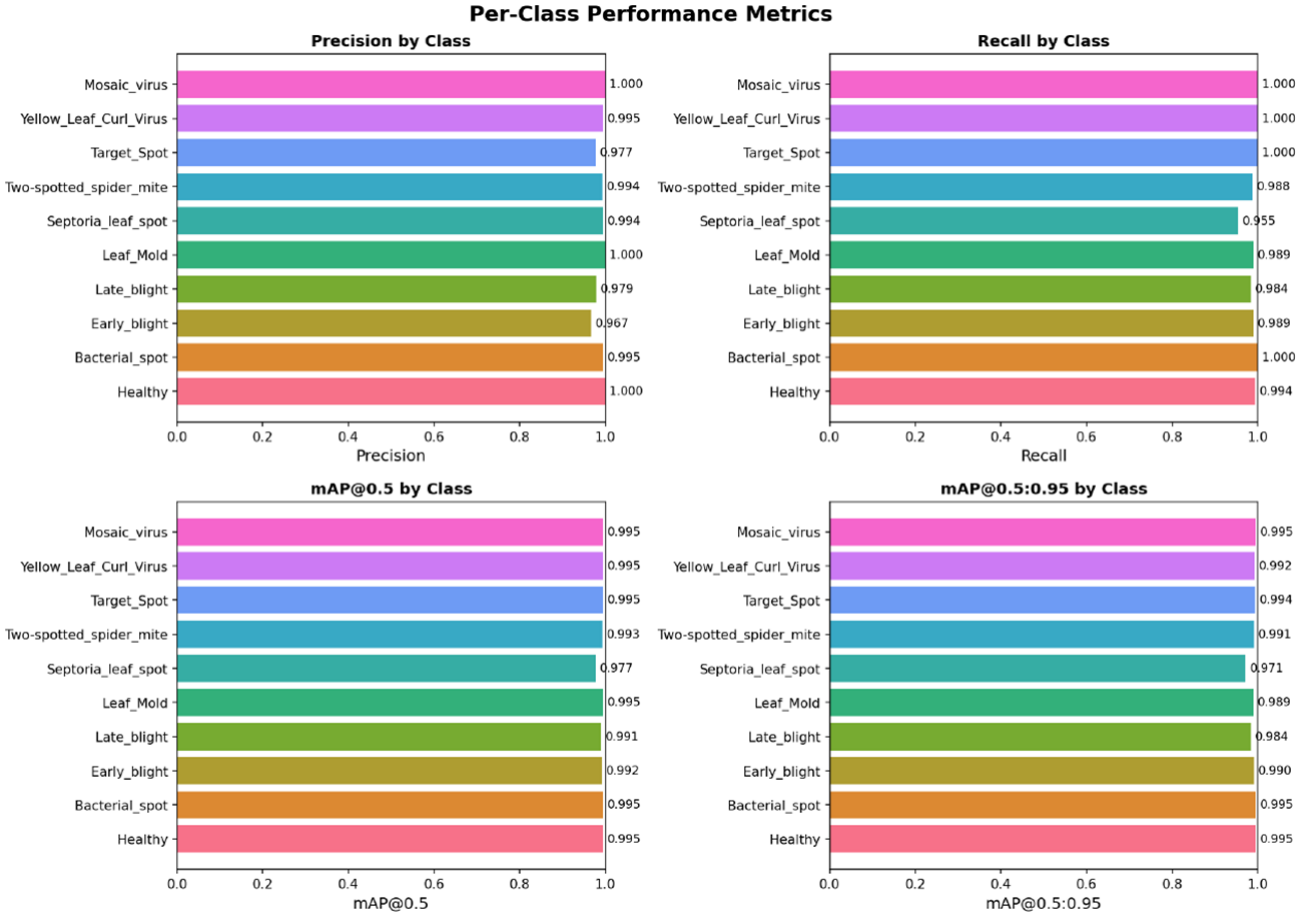
The per-class performance of the YOLOv12 model is reported quantitatively in terms of precision, recall, mAP@0.5, and mAP@0.5–0.95 for ten categories of tomato leaf diseases.

It presents the class-wise performance of the YOLOv12 model evaluated on the Tomato Leaf Disease dataset using precision, recall, mAP@0.5, and mAP@0.5–0.95 **Figure 15**. The model also has high precision and recalls for all the disease types, implying that the localization is accurate and the classification is reliable. The higher mAP scores also verify the robustness of the proposed method for detecting multiple tomato leaf diseases. To sum up, the results indicate both the high discrimination between individual classes and the good ability to generalize well of the proposed YOLOv12 model.

#### 3.2.2 Performance evaluation on the soybean leaf disease dataset

**Figure 16:**
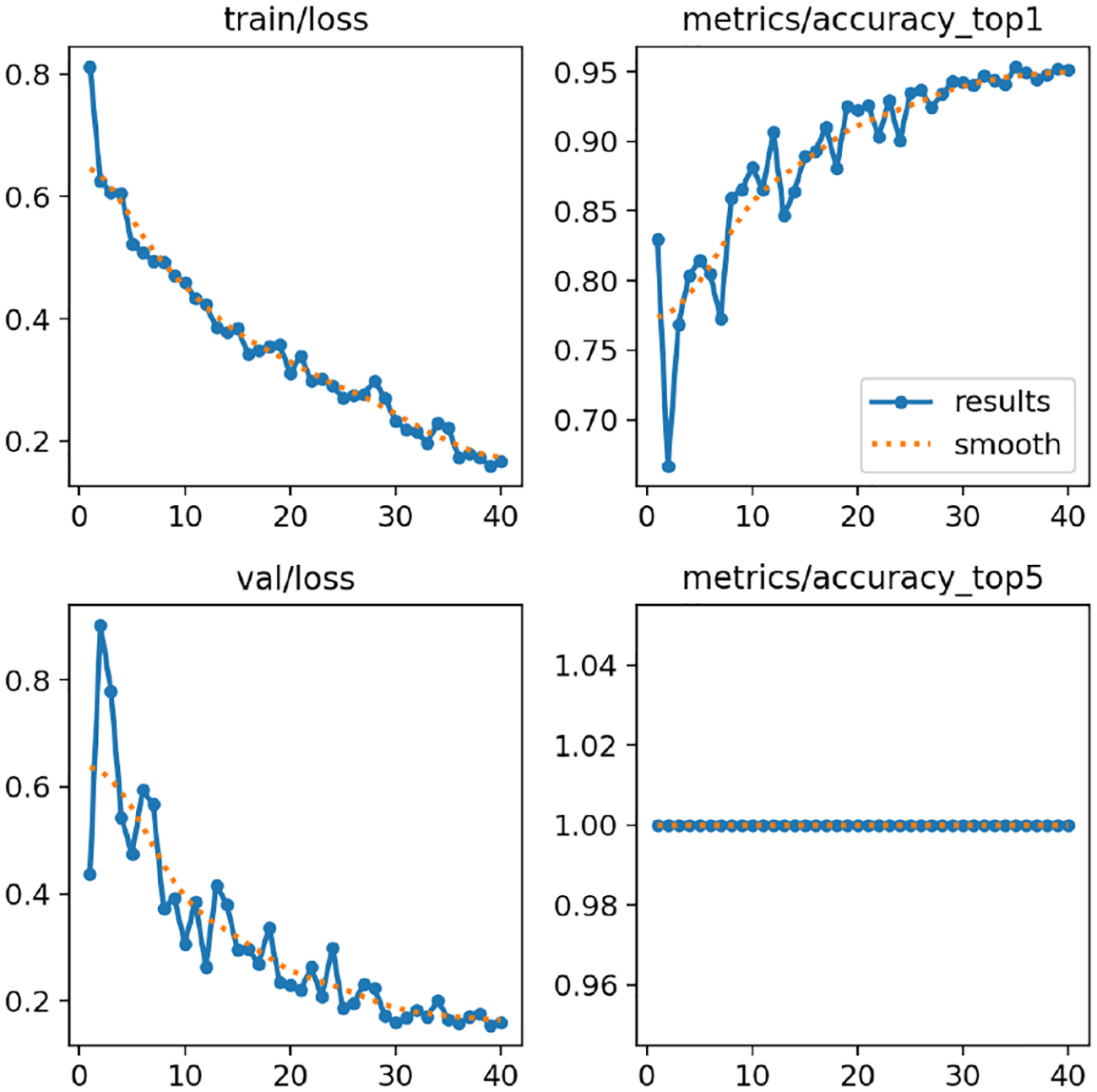
Training loss and accuracy curves of the proposed YOLOv12 model on the soybean leaf disease dataset.

**Figure 16** shows the training and validation loss curves and accuracy curves for the training of the YOLOv12 model on the Soybean Leaf Disease dataset. The training and validation loss follow a similar decreasing pattern, which is a sign of a stable optimization process, and the learning is going well. Their accuracies are increasing steadily and stabilize early, which shows the convergence of the models is reliable. Such trends indicate that the proposed YOLOv12 model can capture discriminative features of soybean leaf diseases while generalizing well in the absence of overfitting.

**Figure 17:**
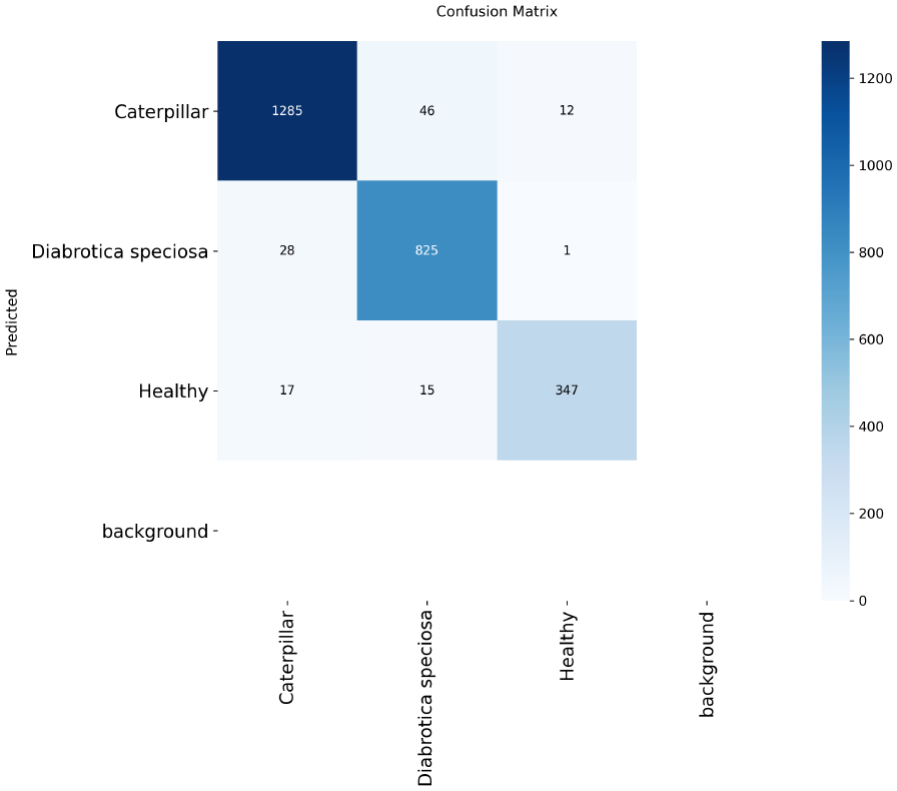
Confusion matrix of the YOLOv12 model.

**Figure 18:**
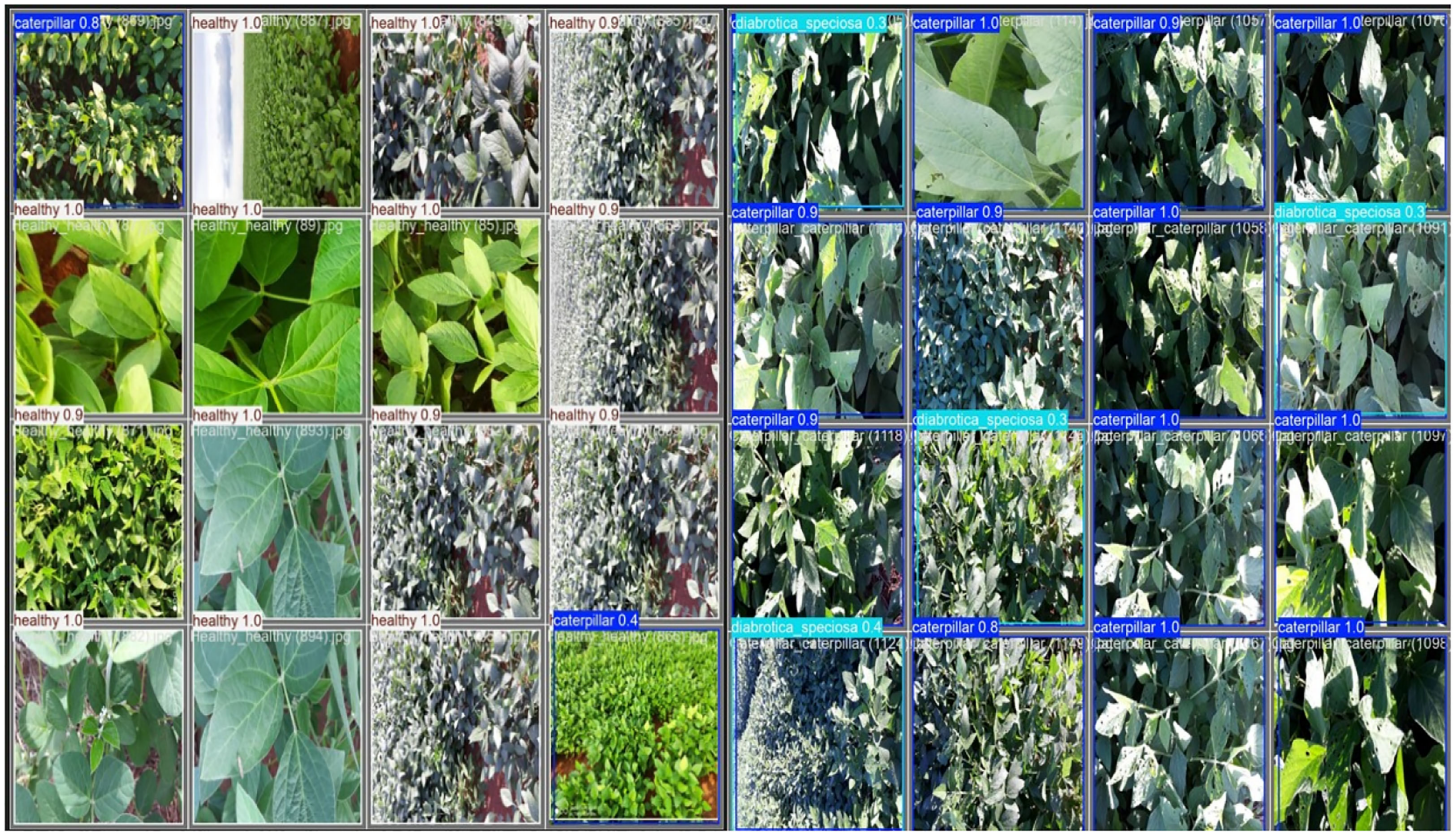
Qualitative detection results of the YOLOv12 model on the soybean leaf disease dataset. The images illustrate bounding-box localization and class prediction across diverse backgrounds and lighting conditions.

**Figure 19:**
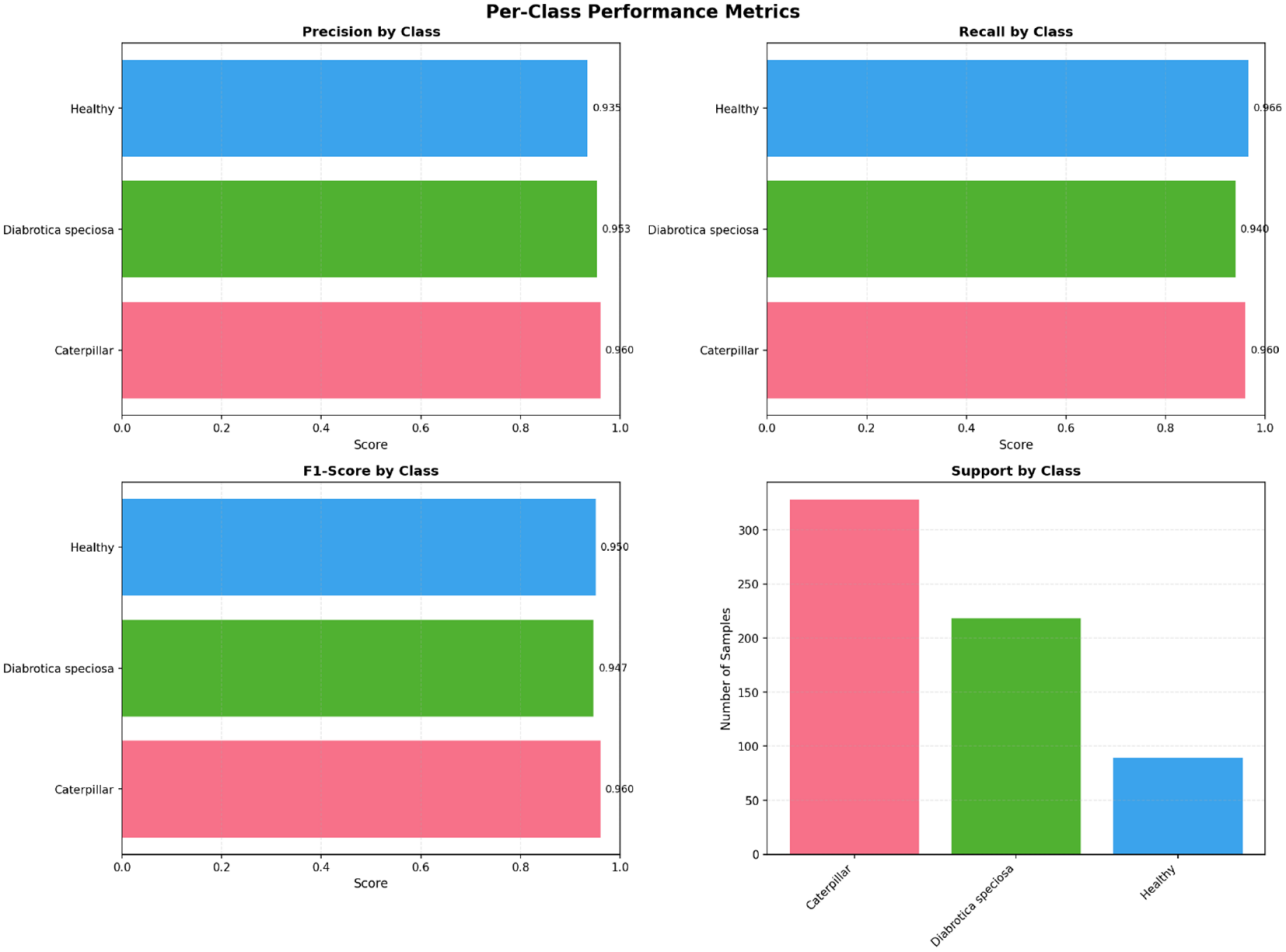
The per-class performance of the YOLOv12 model is reported quantitatively in terms of precision, recall, F1 score and support class for three categories of soybean leaf diseases.

The confusion matrix shows the performance of the YOLOv12 model on the Soybean Leaf Disease dataset with the three disease classes and the background class in **Figure 17**. The model is clearly diagonally dominant, which means it correctly classifies the majority of Caterpillar, Diabrotica speciosa, and Healthy instances. Small misclassifications occur mainly between look-alike categories; background confusion is limited. On the whole, the results demonstrate the effectiveness of our proposed YOLOv12 model to identify soybean leaf disease states in real field images. The qualitative detection results also demonstrate that the YOLOv12 model can well.

localize and classify the soybean leaf disease cases under different field environments (**Figure 18**). The model correctly classifies diseased and healthy areas in the face of differing lighting conditions, background noise, and leaf orientations. This persistence of predicted bounding boxes in target regions indicates that the proposed method is robust. These qualitative results complement the quantitative results and demonstrate that the model can be trusted on real applications too. **Figure 19** shows the precision, recall, F1-score, and class-wise support of the YOLOv12 model on the soybean leaf disease dataset. The model has good precision and recalls for all classes, which provides both precise and reliable localization of diseased and normal soybean leaves. The excellent F1 scores also verify the model has balanced performance that performs well in both false positives and false negatives.

While there are evident disparities for sample sizes among classes, the YOLOv12 model achieves competitive performance for the minority classes and majority classes, demonstrating that it is robust to class distributions bias. The similar detection accuracy for visually similar disease categorize also implies that the model has learned discriminative spatial patterns and texture features. These results overall confirm that the proposed YOLOv12 model is suitable for real-time detection of soybean leaf diseases on an application field conditions, making it useful for practical application in agriculture.

### 3.3 Performance evaluation on tomato and soybean leaf datasets

The detailed quantitative analysis of both the proposed and baseline DL models for the tomato leaf disease dataset over various classification, agreement, and reliability measures. Along with conventional performance metrics such as accuracy, precision, sensitivity, and F1-score, class-wise consistency, prediction confidence and robustness under class imbalance are evaluated by advanced metrics, that is, Matthews correlation coefficient, Cohen’s Kappa, balanced accuracy, log loss, Hamming loss, F2-score, Jaccard index, and mAP@0.5 as show in **Table 7**. This multi-faceted evaluation offers an extensive and unbiased analysis of single CNN models, hybrid models, and detection-based schemes, providing a straightforward determination of their potential to stably and accurately recognize tomato leaf diseases under such varying visual conditions. Comparative results for the tomato leaf dataset are reported, and results show all models reached a high accuracy with some obvious improvements from soybean dataset since visual disease patterns were clearer. MobileNet achieved an accuracy of 96.25%, an efficient and lightweight baseline model. The ResNet152V2 achieved the better result with the accuracy of 99.00%, and the MCC is 0.9929, suggesting very trustworthy classification results. InceptionV3 produced almost flawless performance with 99.14% accuracy, precision, recall and F1-score, also with very low log loss (0.0271) and Hamming loss (0.0056), indicating confident and stable prediction. The proposed hybrid CNN again leads to a strong result, achieving an accuracy of 99.48% with the MCC of 0.9802 and Kappa of 0.9801, thus showing its stability and robustness. The DenseNet121 also yielded a good performance with an accuracy of 97.38% and an F1-score of 97.90%. In addition, the YOLOv12 detection network achieved a good mAP@0.5 of 0.9922, which confirms its superiority in precisely locating diseased areas on tomato leaves. The results show that in terms of classification and detection, our proposed hybrid CNN performs best and most consistently, so it is promising for providing reliable tomato leaf disease diagnosis in real applications.

**Table 7:** Comprehensive evaluation metrics for tomato leaf dataset.

Model	Acc	Prec	Recall	F1	MCC	Kappa	Bal. Acc	LogLoss	Hamm.	F2	Jaccard	mAP@0.5
MobileNet	0.9625	0.9633	0.9625	0.9623	0.9575	–	–	–	–	–	–	–
ResNet152V2	0.9900	0.9902	0.9938	0.9938	0.9929	–	–	–	–	–	–	–
InceptionV3	0.9914	0.9944	0.9944	0.9944	–	0.9936	0.9939	0.0271	0.0056	0.9944	0.9889	–
Hybrid CNN	0.9948	0.9847	0.9745	0.9790	0.9802	0.9801	0.9745	0.1981	0.0182	0.9762	0.9595	–
DenseNet121	0.9738	0.9654	0.9789	0.9790	–	–	–	–	–	–	–	–
YOLOv12	–	0.9906*	0.9872*	–	–	–	–	–	–	–	–	0.9922

The performance of all the tested models on the soybean leaf data, which contains pest damage as well as natural background variation and class imbalance, is shown in **Table 8**. The metrics are standard and reliability based to capture not only the predictive correctness but also the reliability of the model and agreement beyond chance. The table also shows how well some architectures generalize to more challenging and true-to-life field settings, where ensembles and attention-guided methods help suppress background noise and focus on biologically meaningful leaf patterns. This evaluation confirms the robustness and practical viability of proposed framework for identifying pests and diseases from field captured images of soybeans. This assessment establishes the practicality and strong applicability of the proposed system for pest and disease identification from field-taken images of soybean. All of the considered models achieved good classification performance on the soybean leaf dataset, verifying the potential of deep learning architectures for soybean disease identification. MobileNet gave a good baseline result, with an accuracy, precision, recall and F1-score of 96.0%, indicating stable and reliable predictions regarding the macro-averaged measurements. ResNet152V2 enhanced the classification performance to a certain extent, obtaining a high accuracy of 92.82% and a large MCC value of 0.8812, which indicated that the proposed method was capable of making dependable predictions even if the classes were unbalanced. The performance is further improved with InceptionV3 yielding a consistent 94.0% for accuracy, precision, recall and F1-score, which indicate the evenly distributed knowledge learnt for all disease classes. DenseNet121 also demonstrated competitive performance against state-of-the-art methods, with an accuracy of 91.42% and a MCC of 0.8589, proof of efficient feature reusing. Significantly, our proposed Hybrid CNN surpasses other competing baselines, with the best accuracy rate of 96.6%, and also the best precision (89.97%), recall (91.45%) and F1-score (90.48%). Also, Hybrid CNN achieves the best MCC (0.8535) and Kappa (0.8529), which demonstrates strong coincidence. Extensive experiments on a benchmark dataset reveal the effectiveness of the newly proposed framework. Its balanced accuracy (91.45%), low hamming loss (0.0934), and high Jaccard index (0.8262) also indicate the robustness and the generalization ability of the proposed approach to the classification of soybean leaf disease.

**Table 8:** Comprehensive evaluation metrics for soybean leaf dataset.

Model	Acc	Prec	Recall	F1	MCC	Kappa	Bal. Acc	LogLoss	Hamm.	F2	Jaccard	Notes
MobileNet	0.9600	0.9600	0.9600	0.9600	–	–	–	–	–	–	–	Macro Avg = 0.96
ResNet152V2	0.9282	0.9302	0.9282	0.9285	0.8812	–	–	–	–	–	–	Weighted Metrics
InceptionV3	0.9400	0.9400	0.9400	0.9400	–	–	–	–	–	–	–	Macro Avg = 0.94
DenseNet121	0.9142	0.9165	0.9142	0.9145	0.8589	–	–	–	–	–	–	Weighted Metrics
Hybrid	0.9666	0.8979	0.9145	0.9048	0.8535	0.8529	0.9145	3.3651	0.0934	0.9102	0.8262	Macro Metrics

Even though the soybean hybrid achieved a very high 96.66% overall accuracy, accuracy itself can be deceptive in the context of class imbalance, as the DepohtoriaCaterpillar class (*n* = 3,309) overwhelms the dataset. Hence, the macro-averaged precision, recall, and F1-score were regarded to be more trustworthy indicators of performance as show in **Table 9**. The per-class F1-scores (Healthy: 0.8850, DepohtoriaCaterpillar: 0.9299, Diabrotica speciosa: 0.9035) show that the performance was consistently high for all classes. The macros-F1 score also indicates a well-balanced prediction performance without over-prediction on the majority class. In addition, the balanced accuracy of 0.9145 indicates a robust recall in all the classes. Therefore, macro-averaged metrics and balanced accuracy seem to give a better assessment than the overall one in the presence of class imbalance.

**Table 9:** Class-wise precision, recall, and F1-score for the hybrid model.

Class	Precision	Recall	F1-score	Support
Healthy	0.8800	0.8900	0.8850	896
DepohtoriaCaterpillar	0.9200	0.9400	0.9299	3309
Diabrotica speciosa	0.8937	0.9135	0.9035	2205

The training setups and hyperparameters of all the DL models are given in **Table 10** (batch size, learning rate, epochs, the choice of optimizer, the resolution of the input, and model-specific options such as pretraining, finetuning, integrating attention, and detection thresholds). These settings were adopted as a compromise between stable learning, fast convergence, computational efficiency, and fairness of comparison between architectures. Reporting these settings confirms experimental transparency, the ability to reproduce outcomes, and the methodological rigor, as a consequence, enhancing the trustworthiness of the reported performance.

**Table 10:** Hyperparameters across all the DL models used in the current study.

Model	Batch size	Learning rate	Epochs	Optimizer	Input size	Special settings
MobileNet	32	0.001	50	Adam	224 × 224	Pretrained = Yes
ResNet152V2	32	0.0001	50	Adam	224 × 224	Transfer learning enabled
InceptionV3	32	0.0001	50	Adam	299 × 299	Fine-tuned last layers
Hybrid CNN (VGG19+CBAM)	16	0.0001	60	Adam	224 × 224	CBAM + custom dense layers
DenseNet121	32	0.0001	50	Adam	224 × 224	Pretrained; last block tuned
YOLOv12 (Detection)	16	0.00001	100	SGD	640 × 640	Confidence = 0.25, IoU = 0.45, NMS = Enabled

### 3.4 Cross-dataset evaluation

To assess the proposed model’s robustness and practicality in a real scenario, the cross-dataset validation has been performed on the PlantDoc dataset. For this evaluation, only the tomato-related classes were selected from the dataset, namely, tomato Septoria leaf spot, tomato leaf late blight, tomato leaf, tomato leaf mosaic virus, tomato mold leaf, tomato early blight leaf, tomato 835 leaf yellow virus, and tomato leaf bacterial spot. Unlike laboratory-controlled datasets such as PlantVillage, the images in PlantDoc were taken directly in the agricultural fields under natural conditions. These images include large variations in lighting, background clutter, camera tilt, occlusion from overlapping leaves/branches, and other natural phenomena. These features make PlantDoc a very challenging benchmark and a suitable one to test the real-world generalization performance of plant disease detection models.

For this test, only the tomato classes from the PlantDoc dataset were extracted and utilized for testing. These are eight disease classes for well-known tomato leaf diseases in field situations. The images in the dataset are of varying spatial resolution, and we have resized them to 224 × 224 pixels during preprocessing to match the model input size. This evaluation across the datasets allows one to test the model performance under realistic farming conditions with quality images having different illumination and other differences(occlusion) from controlled photograph datasets. We have used the same hyperparameters for evaluating the current dataset to maintain consistency and ensure a fair comparison with the results obtained from the primary training dataset.

#### 3.4.1 Yolo results on cross-dataset validation

**Figure 20:**
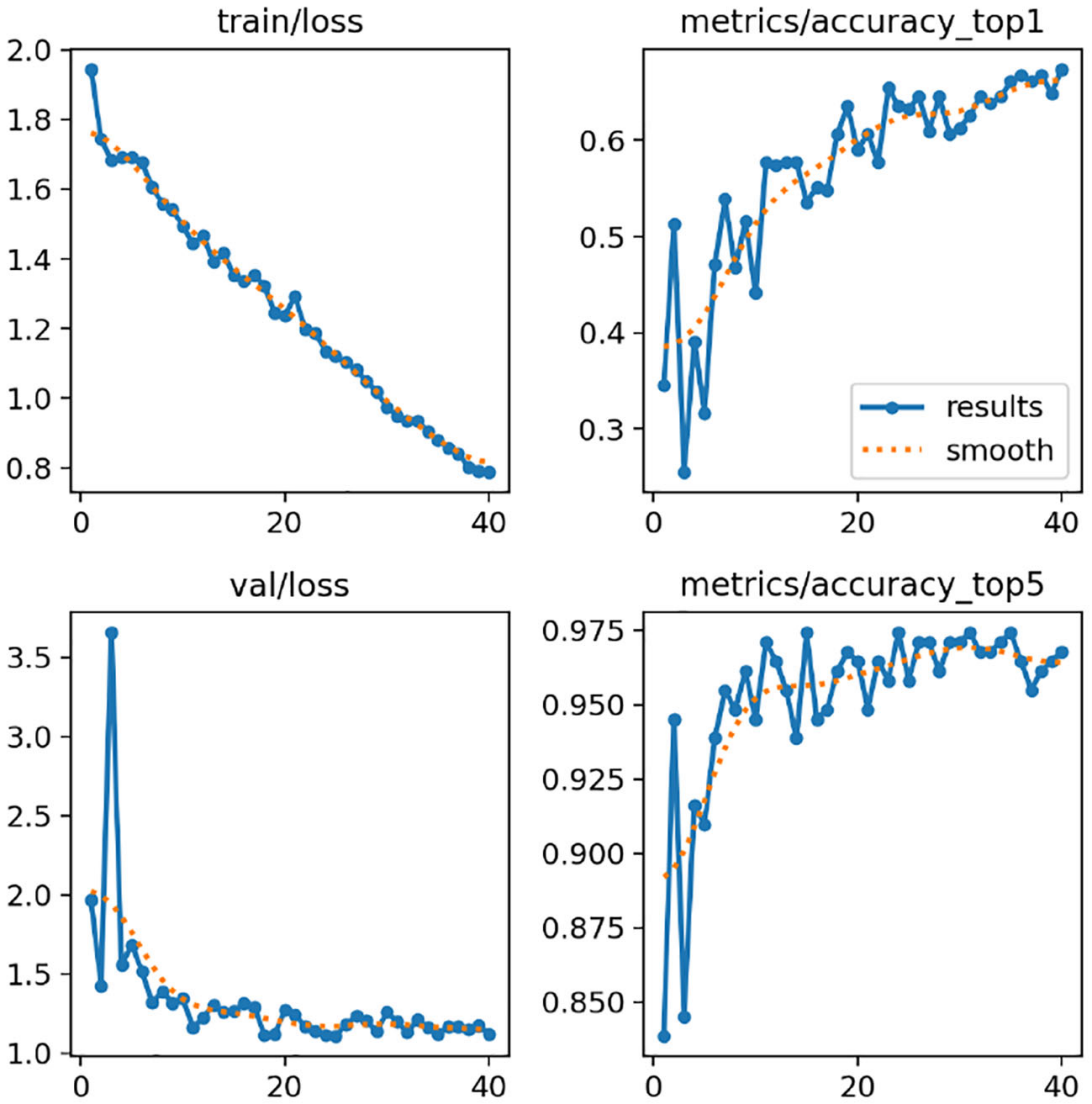
Training and validation performance on the PlantDoc dataset.

The training and validation accuracy curves for cross-dataset validation on the PlantDoc dataset show that the YOLO-based classification model experiences stable learning. As shown in **Figure 20**, the training loss monotonically decreases from about 1.9 to 0.78 in 40 epochs, which proves the valid optimization, and the features learned from the image of tomato leaf are gradually enhanced. The pattern of validation loss is similar, decreasing (initial) and then stabilizing around 1.1, which indicates that although the model is struggling with the full complexity of real-field conditions, including background clutter, illumination variation, and partial occlusions, it is generalizing reasonably. The top-1 accuracy steadily increases during training and is about 66%, whereas the top-5 accuracy is about 96%–97%, which means that the model can find the correct target class in the top several candidate predictions. These results demonstrate that the proposed method achieves comparable or even better results than the state of the art. This result verifies that the performance of our method is not diverged when it is tested on another real-world dataset and under practical agricultural imaging conditions.

**Figure 21:**
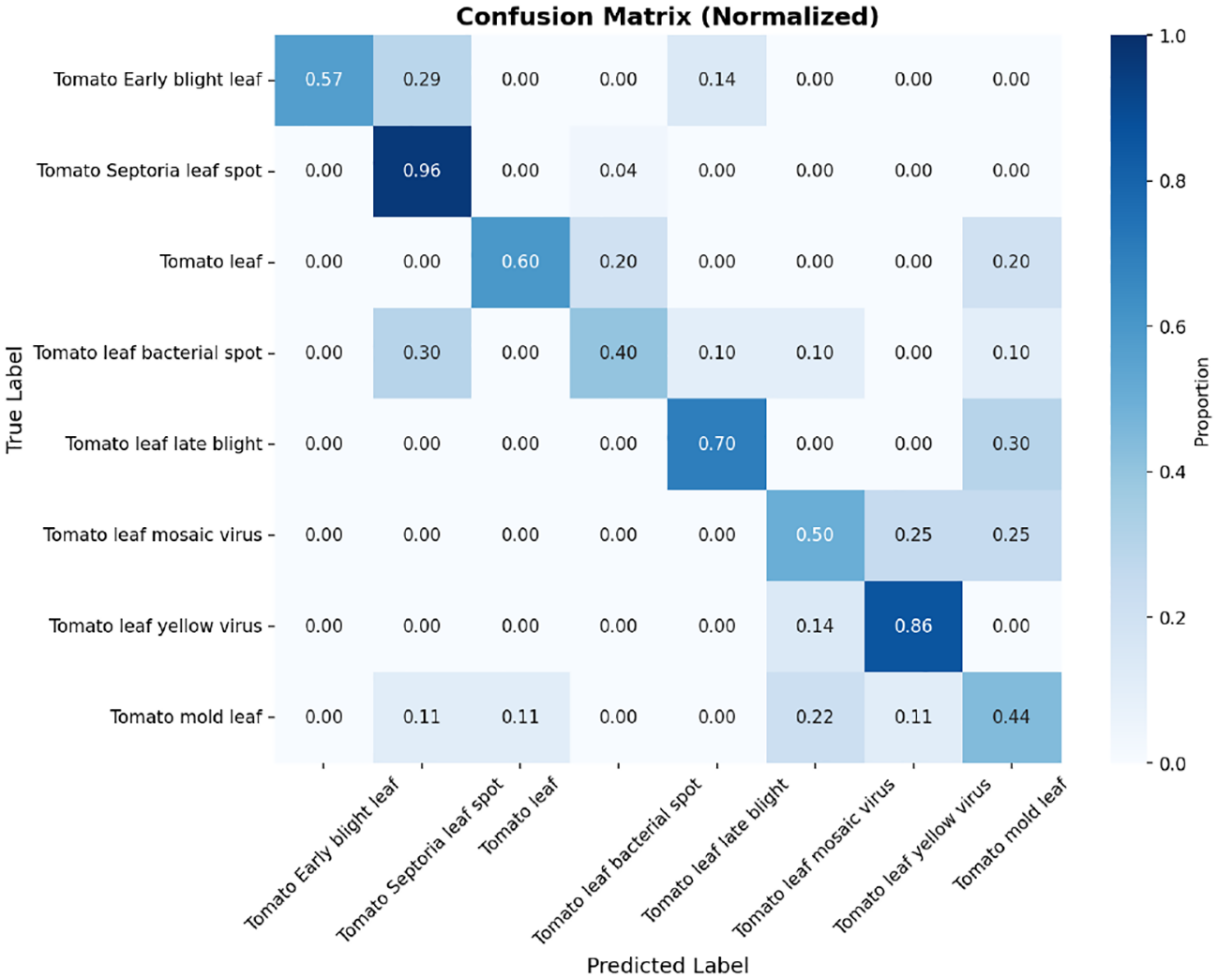
Normalized confusion matrix for the eight tomato leaf classes on the PlantDoc dataset.

The results obtained from cross-dataset evaluation on PlantDoc Dataset is given by a normalized confusion matrix which allows us to analyze the proposed model performance on each class in.

the case of eight tomato leaf classes. As shown in **Figure 21**, the model is well discriminated on several disease categories, in particular on tomato Septoria leaf spot and tomato leaf yellow virus, with the correct identification rate of 0.96 and 0.86, respectively. Similarly, the model has a fairly good identification of tomato leaf late blight with a 0.70 accuracy. On the other hand, a certain level of confusion is observed when comparing the visually similar disease patterns, such as tomato early blight leaf, tomato leaf bacterial spot, and tomato mold leaf, which display similarity in texture and discoloration when observed in a real-field environment. These sorts of misclassifications are to be expected owing to the difficulty of the PlantDoc dataset, whose images feature complex backgrounds, lighting changes, and occlusions. However, the confusion matrix results show that the suggested approach is able to retain good class-separability and has a promising generalization ability when tested with images of field crops.

**Figure 22:**
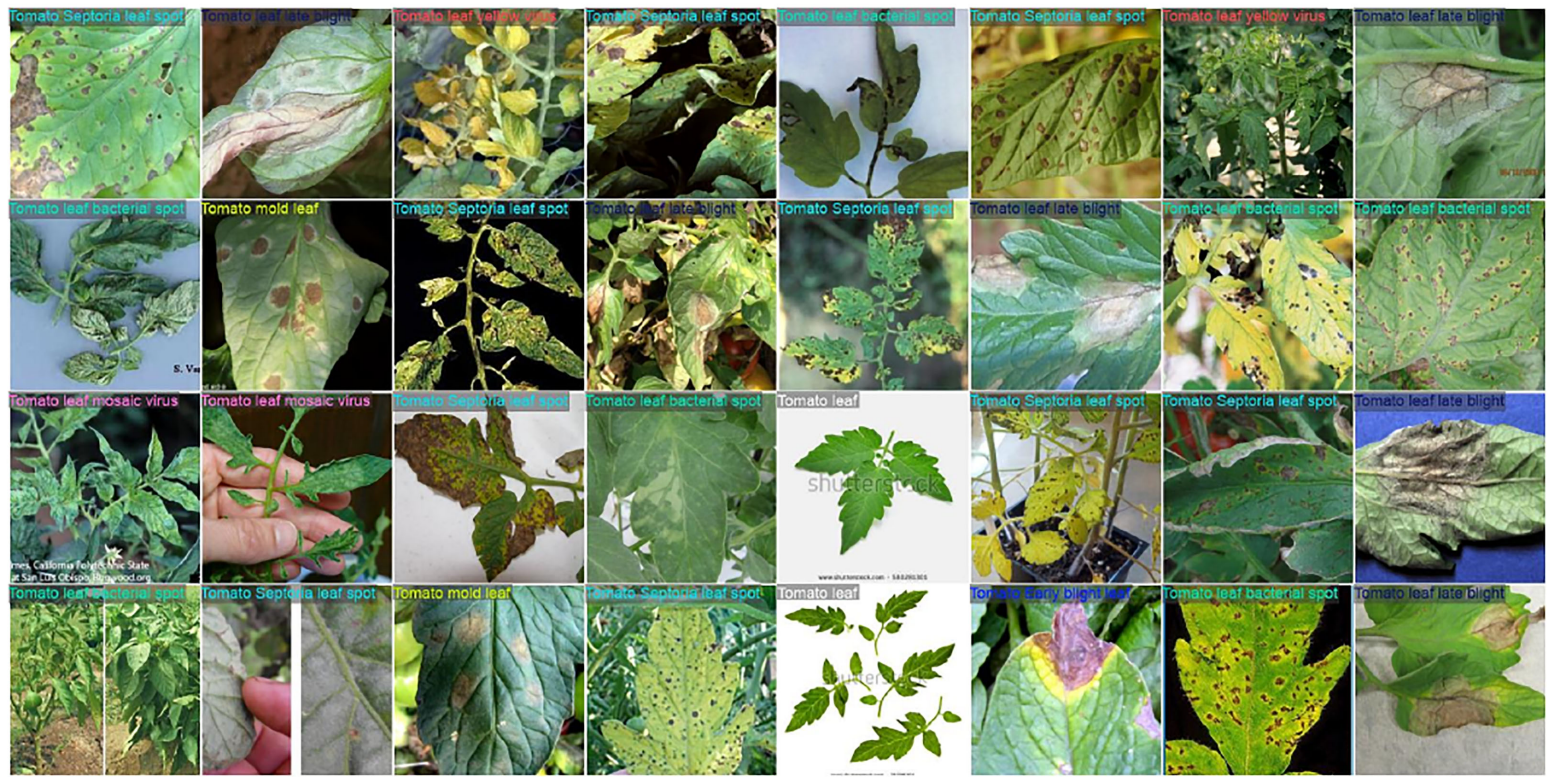
YOLO-based detection results on PlantDoc tomato leaf images showing predicted disease classes under diverse real-field conditions.

**Figure 22** displays a few typical sample images from the PlantDoc Dataset for cross-dataset evaluation. The figure shows various examples of tomato leaf states from the chosen classes, such as tomato Septoria leaf spot, tomato leaf late blight, tomato leaf yellow virus, tomato leaf bacterial spot, tomato leaf mosaic virus, tomato mold leaf, Tomato Early blight leaf, and tomato leaf. These photographs are taken in natural agricultural fields and have a dramatic difference in lighting, background complexity, leaf orientation, and some of them are partially occluded by other plants. Such variance emulates real field conditions, which further challenges automatic disease recognition systems, and thus can be regarded as a reliable test for the modeling generalization.

**Figure 23:**
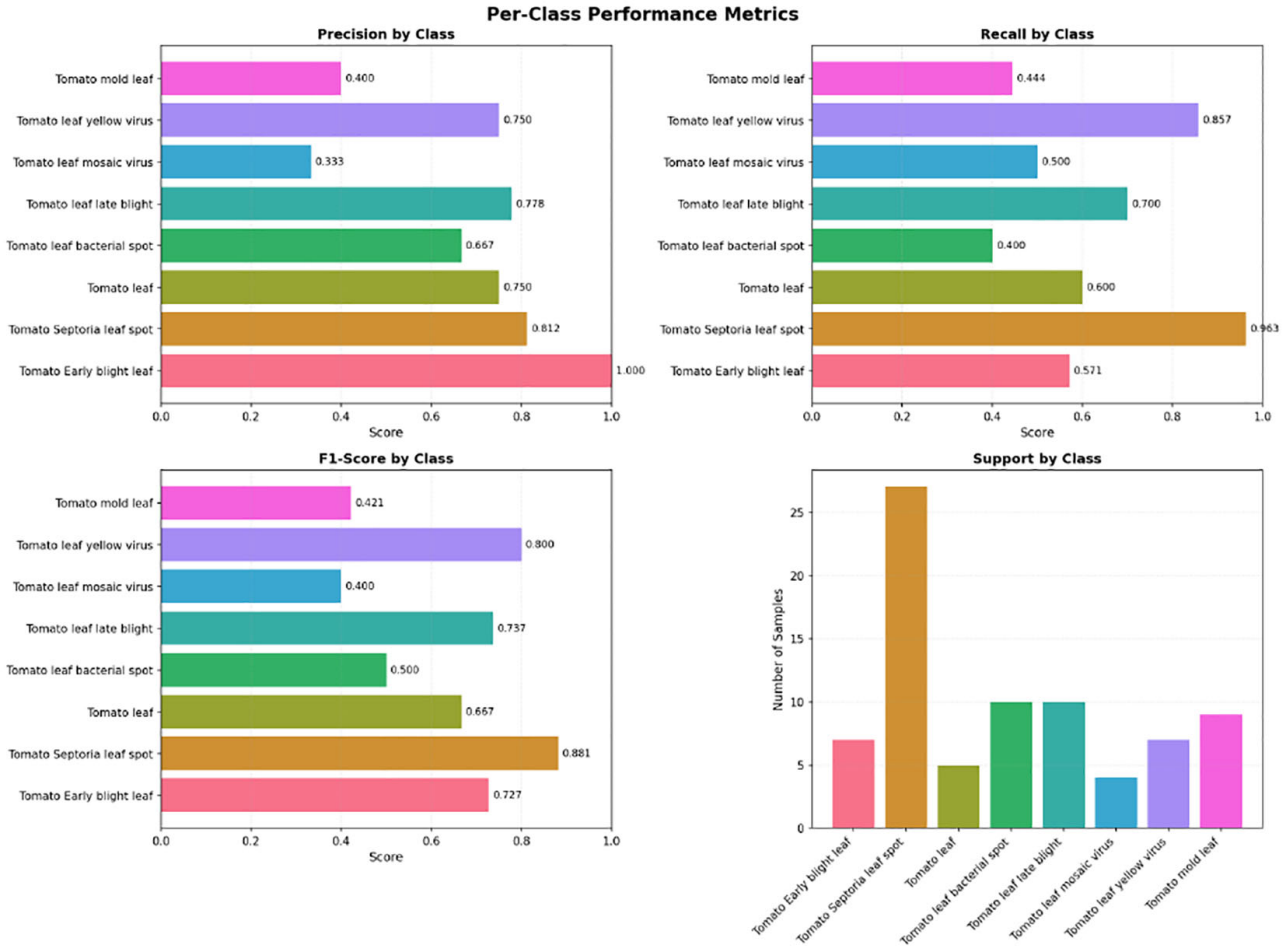
Per-class precision, recall, F1-score, and support for the eight tomato leaf classes on the PlantDoc dataset.

**Figure 23** shows the performance metrics for each class in the cross-dataset evaluation on the PlantDoc Dataset with the retained categories of tomato leaves. The results show that the classification performance of different disease classes differs due to the diversity of visual patterns and the number of samples. Interestingly, tomato Septoria leaf spot and tomato leaf yellow virus are able to yield relatively good results with higher values of precision, recall, and F1-score as shown in **Table 11**, which means that the model can effectively discriminate features. Some classes are at the bottom with lower scores, such as tomato leaf mosaic virus and tomato mold leaf and this is because of the very fewer numbers of samples, and there are some very similar symptoms under complicated field conditions. The distribution of support also reveals the class imbalance, which impacts the performance of each class regardless of the overall view. Nonetheless, these results confirm that the model can still attain acceptable classification performance for several tomato leaf diseases from *in situ* images of agricultural products.

**Figure 24:**
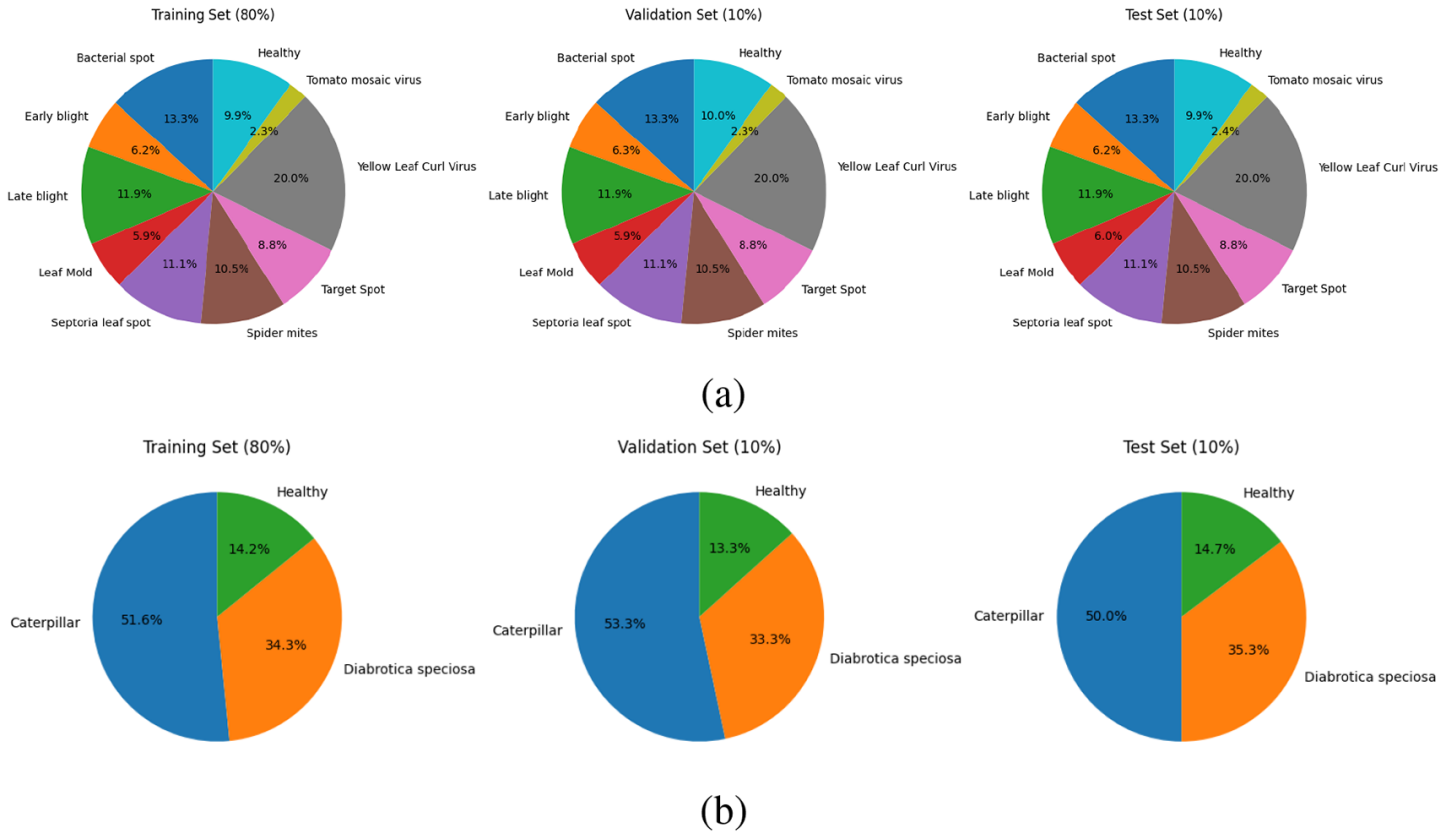
Dataset splitting for leaf images. **(a)** Tomato leaf dataset **(b)** Soybean leaf dataset.

**Figure 25:**
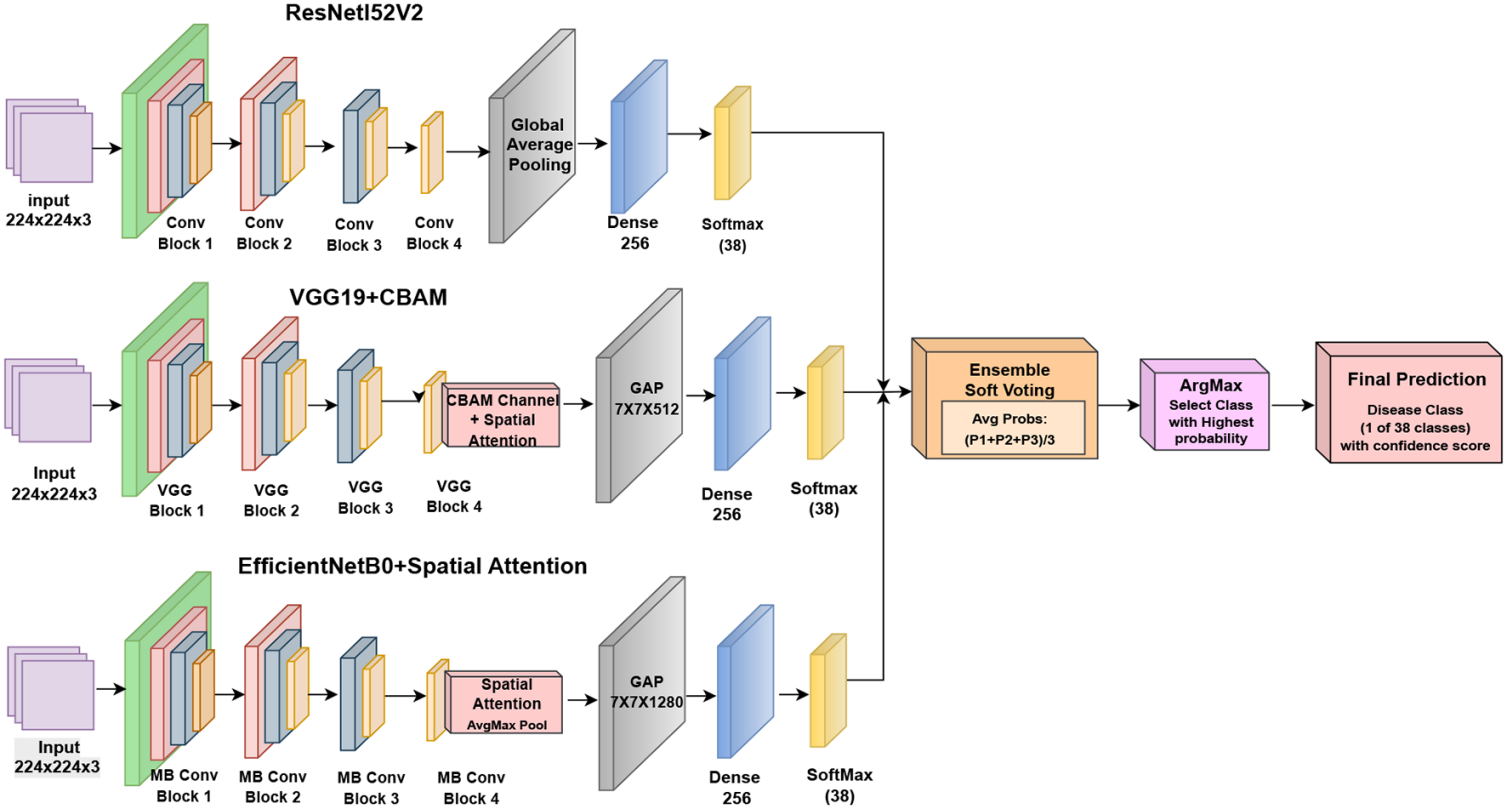
Architecture diagram of the proposed hybrid attention-based stacking ensemble model showing parallel backbone networks (ResNet152V2, VGG19+CBAM, EfficientNetB0 with Spatial Attention) and probability-level soft voting.

**Figure 26:**
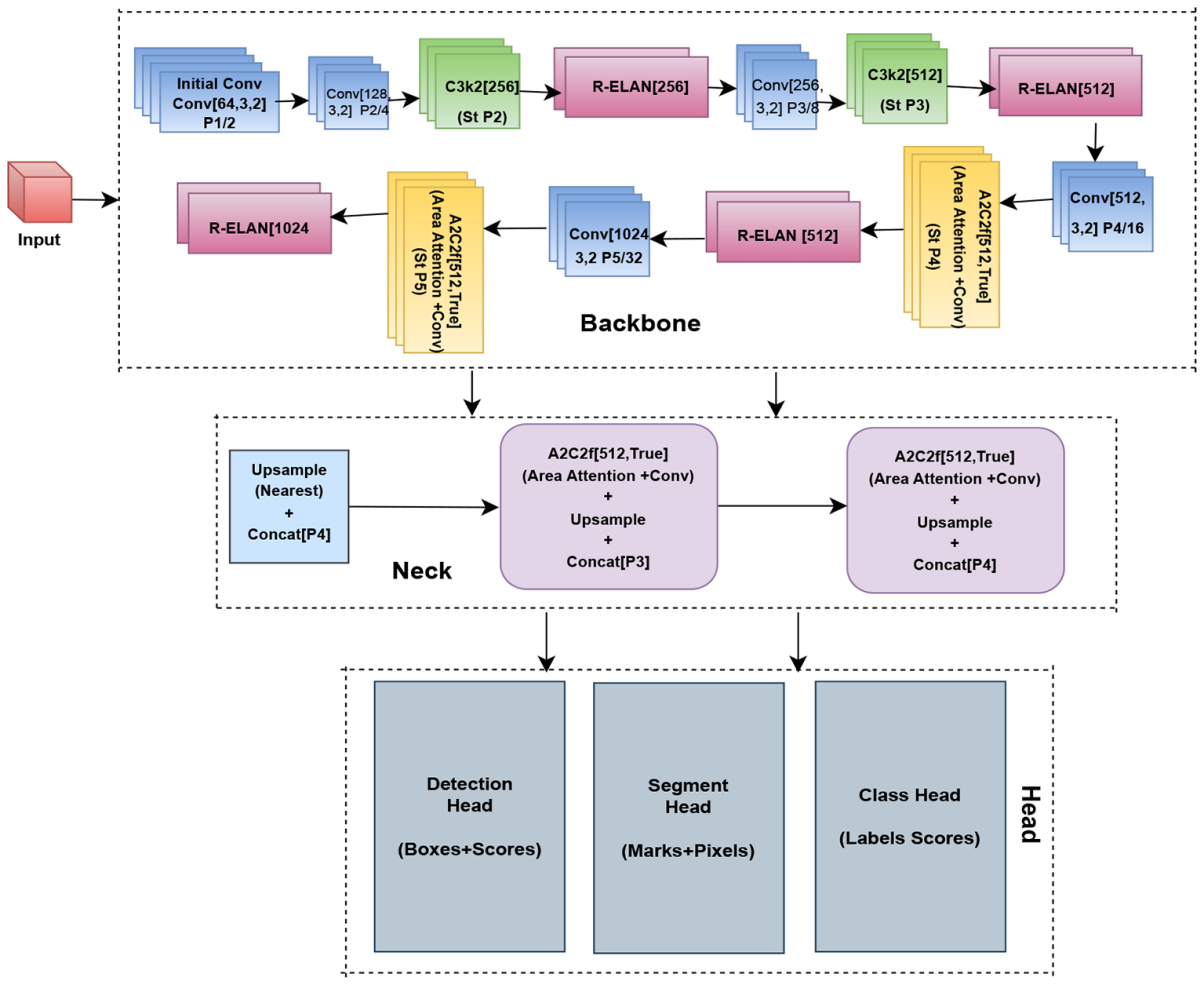
Architecture diagram of YOLOv12.

**Table 11:** Performance comparison on the dataset.

Model	Accuracy	Precision	Recall	F1-Score
MobileNetV2	0.62	0.64	0.62	0.61
InceptionV3	0.72	0.75	0.60	0.68
DenseNet121	0.60	0.61	0.59	0.60
ResNet152V2	0.57	0.59	0.58	0.56
Hybrid Ensemble	0.75	0.72	0.67	0.69

The textural leaf features of eight tomato leaf disease categories in the PlantDoc Village dataset were used to train a YOLO-based detection model, which was tested in the results of **Table 12** with expected results. In general, the model attains good detection results on most classes; a few categories (e.g., tomato leaf late blight, tomato leaf yellow virus) have both low precision and recall and mAP scores, indicating that it is hard to accurately localize and recognize those objects. It is worthy to note that the tomato leaf late blight class reaches almost perfect detection performance with a mAP@50 of 0.995 and a mAP@50–95 of 0.995. In addition, the mAP@50 for healthy tomato leaf is 0.974, which clearly represents that the model can well classify between diseased and healthy leaves. Although all the classes attain strong results, slightly lower precision and recall are reported for tomato leaf bacterial spot and tomato mold leaf, which can be explained by the resemblance of some disease symptoms and leaf texture differences. The YOLO-based model evaluated on the PlantDoc dataset (tomato classes only) achieved a top-3 accuracy of 92.40% and a top-5 accuracy of 98.73%, indicating strong multi-class discrimination capability. These results demonstrate that the model reliably identifies the correct tomato disease class within the top predicted categories, highlighting its effectiveness for practical plant disease recognition scenarios. Taking into account the whole findings, it can be concluded that the YOLO-based method is suitable to be a robust tomato leaf disease detector on the PlantDoc Village dataset.

**Table 12:** Performance of YOLO on the PlantDoc village dataset for tomato leaf disease classes.

Class	Precision	Recall	mAP@50	mAP@50-95
Tomato early blight leaf	0.806	0.905	0.917	0.917
Tomato septoria leaf spot	0.696	0.949	0.906	0.905
Tomato leaf	0.781	0.944	0.974	0.974
Tomato leaf bacterial spot	0.664	0.625	0.708	0.706
Tomato leaf late blight	0.922	1.000	0.995	0.995
Tomato leaf mosaic virus	0.785	0.773	0.840	0.840
Tomato leaf yellow virus	0.855	0.913	0.963	0.963
Tomato mold leaf	0.679	0.847	0.820	0.819

### 3.5 Computational efficiency and real-time performance

To demonstrate the real-world relevance of the framework, we also report a comprehensive analysis of computational efficiency, that is, the average inference time per image and the respective frames per second (FPS) for all architectures we evaluated. The timing was using the experimental setup specified in Section 2.8 to keep the models consistent. Out of the analyzed classification backbones, MobileNetV2 builds upon depthwise separable convolutions to further improve performance and is a natural choice for deployment in resource-constrained environments. ResNet152V2 and DenseNet121 are much deeper architectures with larger capacity in terms of number of parameters, which leads to more computations per inference. InceptionV3 follows the form of a multi-scale convolutional network that captures both local and contextual features and has good inference efficiency. The result is that the run-time behavior observed is very much expected from the architectural design principles and parameter size of each network.

**Table 13** summarizes the inference performance of MobileNetV2, ResNet152V2, InceptionV3, DenseNet121, YOLOv12n, and the proposed hybrid attention-based ensemble model. The YOLOv12n detector achieves 17.50 ms per image (57.15 FPS), demonstrating powerful real-time object detection capability for the lesion localization task. Notably, the proposed hybrid attention-based ensemble model, which fuses several backbone networks and attention modules, can still run at the speed of 12.98 FPS with the same hardware configuration. These results indicate that although deeper networks bring more computational complexity, the proposed method is still able to strike a good balance between accuracy and efficiency. Therefore, the claim of real-time feasibility is justified not only for the detection part of YOLO but also for the full classification-localization pipeline, which makes the system applicable for real deployment in smart agriculture applications.

**Table 13:** Inference time and frames per second (FPS) comparison of the evaluated models.

Model	Inference time (ms)	FPS
MobileNetV2	70.37	14.21
ResNet152V2	75.95	13.17
InceptionV3	78.17	12.79
DenseNet121	76.81	13.02
YOLOv12n	17.50	57.15
**Proposed hybrid attention ensemble**	77.05	12.98

## 4 Discussion

The current section of the manuscript presents the comparison of the proposed model with the existing studies, the potential limitations, and the practical implications of the study. The scope for the proposed model in precision agriculture, crop management, and breeding was also discussed.

### 4.1 Comparison with existing literature

The almost-saturated accuracy on tomato leaf disease can also be explained by PlantVillage being a benchmark where leaf images are usually well centered and taken under controlled lighting with minimal background variation. This reduces the ambiguity between classes and enables state-of-the-art deep learning architectures to obtain very high classification results. Similar trends were also reported in the literature using the same benchmark dataset. To avoid the reported performance being artificially boosted, several safeguards were applied in this work. These are deterministic stratified splitting with a fixed random seed, integrity checks to ensure no sample is duplicated or shared with one in the training set, and uniform augmentation policies among all classification models to ensure a fair benchmarking.

Since a number of models achieve almost saturated performance on this benchmark dataset, the gaps among high-performance architectures are relatively small. As a result, statements about model superiority are considered together with the stability of convergence, loss behavior, and other complementary metrics such as MCC and balanced accuracy, and not solely based on marginal gains of accuracy.

A tabularized comparison with current methods is given in **Table 14**, where diverse evaluation metrics on the proposed model performance are shown. Previous works usually adopted single.

stream architectures, such as VGG16, VGG19, ResNet-50, and the like; however, these models are challenged by the intricate fine-grained local lesion pattern and complex background in images taken in the field.

As shown in **Table 14**, traditional classifiers, like the SVM and KNN, achieve a relatively poor accuracy of 78% and 64%, respectively, in comparison to the advanced deep learning methods. Besides, although newer versions of the YOLO family, such as YOLOv8 to YOLOv12, have enhanced real-time detection, their precision and recall in evaluations on few classes suffer from the trade-offs. On the other hand, the proposed unified pipeline YOLOv12-CNN advances (after seamless integration of global contextual and fine-grained spatial attention mechanisms) accuracy to 99.18%, offering a more robust (with higher-level joint contextual and local inferencing) and 967 interpretable solution for multi-crop (disease) diagnostics.

**Table 14:** Comparative analysis of the proposed framework with contemporary deep learning methods for plant disease identification.

Method	Dataset	Cls	Size	CV field imagery		Rec	Prec	F1	Acc
VGG16 (Paul et al., 2023)	PlantVillage	11	224×224	No	No	92%	92%	92%	87%
VGG19 (Paul et al., 2023)	PlantVillage	11	224×224	No	No	89%	89%	89%	89%
Custom CNN (Paul et al., 2023)	PlantVillage	11	224×224	No	No	95%	95%	95%	95%
SVM Nikith et al. (2023)	Soyabean leaf disease	3	N/A	No	No	N/A	N/A	N/A	78%
KNN Nikith et al. (2023)	Soyabean leaf disease	3	N/A	No	No	N/A	N/A	N/A	64%
CNN Nikith et al. (2023)	Soyabean leaf disease	3	N/A	No	No	N/A	N/A	N/A	96%
Deep CNN (He et al., 2024)	Tomato leaf disease	9	196×196	No	No	N/A	N/A	N/A	93.21%
ResNet50 (He et al., 2024)	Tomato leaf disease	9	196×196	No	No	N/A	N/A	N/A	88.49%
DenseNet121 (He et al., 2024)	Tomato leaf disease	9	196×196	No	No	N/A	N/A	N/A	91.96%
RRDN (He et al., 2024)	Tomato leaf disease	9	196×196	No	No	N/A	N/A	N/A	95%
Alex-Net (Shanthi et al., 2024)	PlantVillage	6	256×256	No	No	N/A	N/A	N/A	91.2%
Yolov8 (Ramos and Sappa, 2025)	Tomato-Village	4	640×640	No	Yes	85.8%	92.6%	N/A	N/A
Yolov9 (Ramos and Sappa, 2025)	Tomato-Village	4	640×640	No	Yes	79.5%	87.4%	N/A	N/A
Yolov10 (Ramos and Sappa, 2025)	Tomato-Village	4	640×640	No	Yes	86.5%	94.2%	N/A	N/A
Yolov11 (Ramos and Sappa, 2025)	Tomato-Village	4	640×640	No	Yes	88.4%	94%	N/A	N/A
Yolov12 (Ramos and Sappa, 2025)	Tomato-Village	4	640×640	No	Yes	87.3%	94.7%	N/A	N/A
PCA_WOA (K-fold) (Badiger and Mathew, 2023)	PlantVillage	9	223×223	No	No	75.8%	N/A	N/A	75.8%
DNN (K-fold) (Badiger and Mathew, 2023)	PlantVillage	9	223×223	No	No	83.5%	N/A	N/A	78.8%
Modified CNN (K-fold) (Badiger and Mathew, 2023)	PlantVillage	9	223×223	No	No	85.9%	N/A	N/A	74.7%
DeepCNN (K-fold) (Badiger and Mathew, 2023)	PlantVillage	9	223×223	No	No	86.3%	N/A	N/A	85.8%
Proposed GJ_GSO_DbneAlexNet (Badiger and Mathew, 2023)	PlantVillage	9	223×223	No	No	91.3%	N/A	N/A	91.6%
**Proposed Model**	Soyabean Leaf Disease	10	224×224	No	Yes	91.45%	89.79%	90.48%	96.66%
**Proposed Model**	Tomato Leaf Disease	10	224×224	No	Yes	97.45%	98.47%	97.90%	99.18%

Instead of designing a new detection architecture, this work adopts a recent and efficient object detection framework in the integrated classification–localization framework. The approach contribution of the paper is in the system-level integration with attention-based CNN ensembles and explainable AI rather than in the modification of the underlying YOLOv12 architecture. The methodological contribution of the work is an integrated solution with concentration-based CNN ensembles and explainable AI and not an extension of YOLOv12 architecture. By integrating area attention and Flash-Attention–based routines, the model effectively captures long-range dependencies across the leaf surface. This ensures that small-scale necrotic lesions are not lost during feature downsampling—a common failure point in the single-stream “black-box” CNN models used in previous benchmarks.

Although previous YOLO versions have been the default for agricultural applications, systematic evaluation on the effectiveness of YOLOv12 in tomato leaf disease recognition has started (Ramos and Sappa, 2025). They found that YOLOv11 achieves the best trade-off between accuracy and latency, while YOLOv12n (the nano version) obtains the best overall results for deployment on edge. Our results extend that one by applying the YOLOv12 structure in a hybrid ensemble. In contrast to the single-stage detection strategy of (Ramos and Sappa, 2025), the two-stream YOLOv12 with an attention-based ensemble model in our work achieves finer feature extraction. This can be seen in our accuracy of 99.18%, implying that although YOLOv12 is a good localized detector, its accuracy can be enhanced by leveraging a domain-specific ensemble to discriminate morphologically closely resembling diseases such as bacterial spot and early blight.

In addition, when considering prior work analyzing deep learning models as black-box systems, the application of Grad-CAM–based explainable AI provides visual evidence that the model focuses on biologically relevant features. The heatmaps are found to be exactly on the lesions, necrotic spots, mosaic patterns, and pest-invaded regions that represent the typical pathological signs for the diseases as mentioned in **Table 4**. The ensemble-based classification and explainability make the proposed framework unique in literature and appropriate for real-world agriculture.

Although the classification and detection modules were evaluated separately in experiments, they are designed to operate sequentially on the same input within a classification-localization pipeline rather than as independent benchmarking tasks. In the workflow we propose, image-level disease diagnosis is carried out by CNN-based models, and then YOLOv12-based lesion localization is employed to enable spatially explicit interpretation of symptom regions. The current study deals with algorithmic validation in well-controlled experimental conditions; therefore, statements on practical feasibility should be considered as an indication of system potential and not as that of fully developed end-to-end field implementation. Future work will focus on extending the framework to the full pipeline evaluation using field-collected data and real-time applications in precision agriculture.

In fact, the experimental verifications are only on these two benchmark datasets, though the performance is better on both of the two tomato and soybean datasets among the state-of-the-art methods. Hence, the results show that the proposed framework holds promise for cross-crop adaptability using controlled datasets, but generalization to other crop species, environmental conditions, and imaging devices must be taken with a grain of salt. In the future, it will be validated on other field-acquired multi-crop datasets to further prove its robustness and applicability.

### 4.2 Limitations of the study

Although the performance of the proposed framework is considered strong, this paper still has some limitations. First, the experimental evaluation was performed on the publicly available PlantVillage dataset. Despite the extensive data augmentation to enhance generalization, these datasets may not adequately represent the diversity of real-field scenarios, including complex backgrounds, partial occlusions, varying illumination, and variances of camera quality observed in the field. The validation so far is limited to two publicly available datasets; thus, exhaustive generalization for a broad range of crops and field conditions cannot be guaranteed in this work.

Second, the hybrid attention-based ensemble model enhances the accuracy and robustness at the cost of the additional computing complexity in comparison with lightweight single-model architecture. This may hinder direct use on resource-limited edge devices without additional optimization. To move forward from this limitation, the next phase of this research will involve mobile deployment profiling, that is, evaluating the inference latency (in milliseconds) and the battery consumption of the hybrid ensemble on ARM-based edge devices to determine the need for model pruning or quantization. Also, while the YOLOv12 achieves very good real-time detection and localization results, the performance under extreme field situations like heavy occlusion or overlapped disease symptoms is not yet clear.

In the end, the scope of this research is only tomato and soybean crops. While the method has demonstrated robust performance across different datasets, how well it can be generalized to other crop species, disease types, and large-scale field operations remains uncertain. Future work should be carried out to test the proposed method on different crops and real-field data and also on model compression and optimization for practical implementation.

Finally, for practical feasibility, we plan to perform a small-scale pilot field study with a mobile application prototype to compare the system’s real-time diagnostic outputs with live expert agronomists in a realistic agricultural setting.

While the quantitative performance is strong, it should be noted that plant leaves in benchmark datasets such as PlantVillage do not represent real field conditions, where leaves are often occluded, touched by different illumination, or placed against complex backgrounds. As such, the results obtained should be regarded as indicative of benchmark performance rather than full real-world generalization, illuminating a key path for future work in field-based validation. As a concrete next step to address this, we suggest a cross-domain evaluation study in which existing models will be evaluated on ‘out-of-distribution’ datasets such as PlantDoc or FieldPlant to measure the decay in accuracy as the system is subjected to real-world environmental noise that is non-benchmark.

### 4.3 Biological and practical implications

From the growth rate observation and plant pathology point of view, this study demonstrates the framework’s ability to effectively capture disease-related visual features for the various leaf diseases. The Grad-CAM–based explainability analysis shows that the model nearly always pays attention to biologically meaningful areas like lesions, necrotic and chlorotic spots, vein distortions, and pest-damaged tissues. These are closely related to well-known pathological symptoms of tomato and soybean leaf diseases, which illustrate that the model predictions are not dependent on irrelevant features or spurious backgrounds.

The scientific basis of our model is also supported by the Grad-CAM explainability analysis that suggests strong alignment between model attention and certain pathological markers. For example, the attention of the model on necrotic centers and chlorotic halos on tomato leaves is exactly consistent with the physiological symptom specified by pathological keys for Septoria and Xanthomonas diseases. This indicates that the network is not utilizing “spurious correlations,” such as background soil or lighting, but rather is learning biologically diagnostic features. By establishing that the presence of area attention compels the network to encode such textures, we rebut the “black box” critique of deep learning in agriculture, demonstrating the model follows the same visual diagnostic heuristics as human expert agronomists.

Identification of leaf diseases at early stages is important to reduce the loss of crop production and the dissemination of the disease from the viewpoint of agricultural practice. The good classification accuracy and robust infected region localization obtained from both the hybrid ensemble and the YOLOv12 detector allow for early diagnosis and intervention of the disease. This type of precision can enable precision pesticide applications, decrease unnecessary chemical applications, and help lead to more sustainable crop management.

From a practical viewpoint, the combination of the YOLOv12 detector and the hybrid ensemble provides a precise localized intervention. In the case of soybean crops, distinguishing between damage caused by pests (e.g., Diabrotica speciosa) and foliar diseases allows producers to treat them with targeted products instead of broad-spectrum chemicals. This kind of early and accurate diagnosis enables “site-specific” pesticide application, which has been demonstrated to reduce erroneous application of chemicals and to make crop management more sustainable.

Additionally, the interpretability by attention mechanisms and XAI builds trust and usability for both farmers, agronomists, and agricultural extension officers. By visually marking affected areas on the leaves, the developed system enables users to make informed decisions and contributes to disease evaluation on the field, thus allowing the transfer of state-of-the-art deep learning models to practical applications in agriculture.

### 4.4 Applications in agricultural research, crop management, and breeding

The developed deep learning framework can be used in a number of agricultural applications (e.g., research, crop management, and breeding). In the field of agriculture, the proposed system can be utilized to perform large-scale phenotyping and disease monitoring with high classification accuracy and explainable decision-making. By incorporating XAI, this framework enables researchers to conduct systematic analyses of progression patterns of the disease and severity of symptoms, facilitating data-driven investigations in the field of plant pathology and crop health monitoring.

From a crop management point of view, the integration of classification and detection in real-time using the YOLOv12 model allows real practical application in precision agriculture systems. Localization of diseased regions also enables localized intervention strategies, including site-specific pesticide treatment and early removal of infected plants, which can slow disease spread and reduce pesticide use. Moreover, the system is suitable for incorporation into mobile diagnostic applications and intelligent farm systems to enable farmers and agronomists to make decisions in a timely manner, which may contribute to improved yield stability and sustainable farming.

For plant breeding applications, the suggested method can be efficient for screening for disease resistance in large populations of plants. The framework can also help breeders to assess varietal resistance and susceptibility in a controlled or semi-field environment by providing early accurate detection and localization of disease symptoms. This automated and quantitative evaluation has the potential to reduce time, labor, and subjectivity of conventional visual estimation, leading to faster development of disease-resistant crops.

## 5 Conclusion

Leaf disease has a serious impact on crop production and, therefore, on the world food supply, and intelligent solutions for the diagnosis of the diseases are very much needed. In this research work, we present a deep learning-based generalized framework for plant leaf disease detection and classification on two plant species, tomato and soybean, with a benchmark dataset. Preprocessing, data augmentation, and transfer learning are integrated in the framework to effectively address the challenges of class imbalance, intra-class variability, and complex backgrounds. A comparison of recent CNN architectures demonstrates that deep learning techniques are on-plane superior to the traditional machine learning methods due to the fact that the features learned by CNNs are distinguishable ones by directly taking the raw images as input. DenseNet121, MobileNetV2, and InceptionV3 are considered to have good performance, and the latter achieves the best accuracy for tomato leaves. The combination with YOLO-based object detection can be further developed for the localization of disease areas in order to enable high-speed and high-resolution disease identification. In addition, a framework fusing several deep models with spatial and channel attention mechanisms to improve robustness, generalization, and interpretability. Attention concentrates the network on disease-related regions and suppresses background noise, which contributes to the stability of classification. The proposed model achieves the best results on the two evaluated tomato and soybean data sets. The model also has the potential to be extended to other crops after further verification. Further, explainability via Grad-CAM validates biological meaningful learning of features, which makes the proposed framework an accurate, interpretable, and scalable solution for the intelligent diagnosis of plant diseases, enabling both resource-constrained and high-performance computing applications in precision agriculture.

Future work is geared toward three quantifiable experimental axes that will enable us to advance this framework from a proof-of-concept to a deployable tool. Firstly, a cross-dataset generalization experiment will be performed by evaluating the models trained on this benchmark on “out-of-distribution” datasets, such as PlantDoc and FieldPlant, to provide a baseline on how the model decays in performance due to real-world noise. Second, a hardware-specific profiling analysis will be conducted on mobile devices to assess the accuracy-latency trade-offs of the hybrid ensemble and the pruned single-model counterparts in terms of inference latency and the battery consumption on ARM-based edge devices. Lastly, the study will conclude with a long-term field pilot, contrasting AI-based diagnoses with the assessments of the expert agronomists, to offer a quantitative “trust score” of the system across variable field conditions. This quantifiable perspective ensures the proposed framework will act as a validated stepping stone for autonomous, explainable decision support systems for sustainable precision agriculture.

## Data Availability

The original contributions presented in the study are included in the article/**Supplementary Material**. Further inquiries can be directed to the corresponding author.
